# Biomarkers of cellular senescence and risk of death in humans

**DOI:** 10.1111/acel.14006

**Published:** 2023-10-06

**Authors:** Jennifer L. St. Sauver, Susan A. Weston, Elizabeth J. Atkinson, Michaela E. Mc Gree, Michelle M. Mielke, Thomas A. White, Amanda A. Heeren, Janet E. Olson, Walter A. Rocca, Allyson K. Palmer, Steven R. Cummings, Roger A. Fielding, Suzette J. Bielinski, Nathan K. LeBrasseur

**Affiliations:** ^1^ Department of Quantitative Health Sciences Mayo Clinic Rochester Minnesota USA; ^2^ Department of Epidemiology and Prevention Wake Forest University School of Medicine Winston‐Salem North Carolina USA; ^3^ Robert and Arlene Kogod Center on Aging Mayo Clinic Rochester Minnesota USA; ^4^ Department of Neurology Mayo Clinic Rochester Minnesota USA; ^5^ Women's Health Research Center, Mayo Clinic Rochester Minnesota USA; ^6^ Division of Hospital Internal Medicine Mayo Clinic Rochester Minnesota USA; ^7^ Departments of Medicine, Epidemiology and Biostatistics University of California San Francisco San Francisco California USA; ^8^ Research Institute, California Pacific Medical Center San Francisco California USA; ^9^ Nutrition, Exercise Physiology and Sarcopenia Laboratory, Jean Mayer USDA Human Nutrition Research Center on Aging Tufts University Boston Massachusetts USA; ^10^ Paul F. Glenn Center for the Biology of Aging Research Mayo Clinic Rochester Minnesota USA; ^11^ Department of Physical Medicine and Rehabilitation Mayo Clinic Rochester Minnesota USA

**Keywords:** aging, cohort study, GDF15, inflammation, mortality, senescence‐associated secretory phenotype (SASP)

## Abstract

A robust and heterogenous secretory phenotype is a core feature of most senescent cells. In addition to mediators of age‐related pathology, components of the senescence associated secretory phenotype (SASP) have been studied as biomarkers of senescent cell burden and, in turn, biological age. Therefore, we hypothesized that circulating concentrations of candidate senescence biomarkers, including chemokines, cytokines, matrix remodeling proteins, and growth factors, could predict mortality in older adults. We assessed associations between plasma levels of 28 SASP proteins and risk of mortality over a median follow‐up of 6.3 years in 1923 patients 65 years of age or older with zero or one chronic condition at baseline. Overall, the five senescence biomarkers most strongly associated with an increased risk of death were GDF15, RAGE, VEGFA, PARC, and MMP2, after adjusting for age, sex, race, and the presence of one chronic condition. The combination of biomarkers and clinical and demographic covariates exhibited a significantly higher c‐statistic for risk of death (0.79, 95% confidence interval (CI): 0.76–0.82) than the covariates alone (0.70, CI: 0.67–0.74) (*p* < 0.001). Collectively, these findings lend further support to biomarkers of cellular senescence as informative predictors of clinically important health outcomes in older adults, including death.

## Graphical abstract

Circulating components of the (SASP) have been studied as biomarkers of senescent cell burden. We hypothesized that senescence biomarker levels could predict mortality even in healthy older adults. Increases in GDF15, VEGFA, PARC, and MMP2, and decreases in RAGE, were associated with an increased risk of death. Our results suggest that SASP biomarkers may be informative of biological age and clinically important health outcomes, including death.

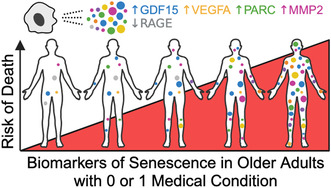

## 1 INTRODUCTION

Cellular senescence is a mediator of aging and chronic disease (LeBrasseur et al., 2015). Diverse forms of age‐related damage, including DNA lesions, mitochondrial dysfunction, proteotoxic aggregates, and inflammation converge on a common cellular fate of stable growth arrest, or senescence (Newgard & Sharpless, 2013). Senescent cells accumulate with advancing age and are enriched in human tissues with disease and/or dysfunction, including skeletal muscle (Englund et al., 2023), lung (Schafer et al., 2017), brain (Neumann et al., 2023), adipose tissue (Choudhery et al., 2014), bone (Farr et al., 2016), and kidney (Barnett et al., 2012). A distinguishing characteristic and mechanism by which senescent cells drive age‐associated pathologies across multiple organs is the senescence associated secretory phenotype (SASP).

The SASP is remarkably heterogenous and differs by cell type. Components of the SASP include cytokines, chemokines, matrix remodeling proteins, growth factors, and a host of other bioactive molecules, such as miRNA and lipids (Coppe et al., 2010; Lopes‐Paciencia et al., 2019; Terlecki‐Zaniewicz et al., 2018). The robust, diverse, and secreted nature of the SASP led to the premise that its components could be leveraged as circulating biomarkers of senescent cell burden. A candidate panel of senescence biomarkers was developed by quantifying the SASP of several human cell types in vitro (Schafer et al., 2020). In humans, the circulating concentrations of candidate senescence biomarkers have exhibited positive associations with chronological age and clinical manifestations of advanced biological age, including, frailty, adverse postsurgical outcomes, and reduced physical function (Fielding et al., 2022; Schafer et al., 2020), in a manner consistent with increased senescent cell burden. Although not unique to senescent cells, the biological diversity of the included proteins, their enrichment in tissues with high senescent cell burden, their inclusion in senescence‐specific gene sets (Coppe et al., 2010; Saul et al., 2022), and their increased circulating abundance in preclinical models with high senescent cell burden (Englund et al., 2023; Yousefzadeh et al., 2020), provide the foundation for their use as biomarkers of senescence.

Our previous studies have largely been cross‐sectional and focused predominantly on persons with established chronic diseases (e.g., aortic stenosis, ovarian cancer, and idiopathic pulmonary fibrosis), frailty, and functional limitations (Aversa et al., 2023; Fielding et al., 2022; Schafer et al., 2020). To advance the utility of senescence biomarkers in humans and to provide further evidence in support of their potential as biomarkers of biological age, we tested the hypothesis that, among relatively healthy older persons with zero or one chronic condition at baseline, those with higher concentrations of senescence biomarkers would have an increased risk of death. To this end, we used the resources of the Mayo Clinic Biobank to study associations between SASP biomarkers and risk of death in approximately 2000 participants over the age of 65 with zero or one chronic condition.

## 2 RESULTS

### 2.1 Study participants

The Mayo Clinic Biobank is an institutional resource comprised of 57,000 adult volunteers, including 24,244 persons over the age of 65 years, who have donated biological specimens, provided risk factor data, and have given permission to access their electronic health records (EHR) for clinical research studies (Olson et al., 2019). We randomly selected eligible participants from the Mayo Clinic Biobank with zero or one chronic condition for this study. One person did not provide consent to use their EHR for research and 72 persons did not have medical records available in the time window of the study or did not have any diagnostic information in their records. Therefore, 1923 persons were included in our final study population.

Characteristics of the study population, including 1066 women and 857 men 65 years of age or older, are shown in Table 1. The study population was predominantly white (97%) and non‐Hispanic (99%). Additionally, 68% of study participants had no chronic conditions at baseline and 32% had one condition. The most common chronic conditions in this population were arthritis (*n* = 208; 10.8%), hyperlipidemia (*n* = 108, 5.6%), and a history of any cancer (*n* = 129; 6.7%; Supplemental Table 1). The median duration of follow‐up was 6.3 years (interquartile range: 1.7, 8.7), and 283 deaths occurred. Cause of death was missing for 74 persons (26.2%), but neoplasms (*n* = 62; 21.9%), diseases of the circulatory system (e.g., cardiovascular diseases; *n* = 41; 14.5%), and diseases of the nervous system (e.g., dementia; *n* = 36; 12.7%) were the most common causes of death in this population. As expected, older age and male sex were associated with an increased risk of death (Table 1). However, persons with one chronic condition at baseline were less likely to die during follow‐up compared to persons with no chronic conditions.

**TABLE 1:** Risk of death by participant characteristics.

Characteristic	*n* (%)	Unadjusted HR (95% CI)	Adjusted HR* (95% CI)
All persons in cohort	1923		
Number of deaths	283 (14.7)		
Age group			
65–69	861 (44.8)	Referent	Referent
70–74	562 (29.2)	1.49 (1.09–2.06)	1.49 (1.08–2.05)
75+	500 (26.0)	3.61 (2.74–4.75)	3.42 (2.59–4.51)
Sex			
Men	857 (44.6)	Referent	Referent
Women	1066 (55.4)	0.50 (0.39–0.64)	0.53 (0.42–0.68)
White race			
No	61 (3.2)	Referent	Referent
Yes	1862 (96.8)	1.07 (0.51–2.27)	1.06 (0.50–2.25)
Hispanic ethnicity			
No	1906 (99.1)	Referent	Referent
Yes	17 (0.9)	0.68 (0.17–2.71)	1.02 (0.25–4.12)
Number of chronic conditions at baseline			
0	1313 (68.3)	Referent	Referent
1	610 (31.7)	0.46 (0.36–0.61)	0.48 (0.37–0.63)

Abbreviations: CI, confidence interval; COPD, chronic obstructive pulmonary disease; HR, hazard ratio; ; TIA, transient ischemic attack.

* Adjusted for age, sex, race, ethnicity, and number of conditions at baseline.

### 2.2 Age, sex, and biomarkers of cellular senescence

The plasma concentrations of 28 candidate senescence biomarkers were measured in plasma samples from the participants (Supplemental Table 2). Twenty‐three biomarkers (82%) were significantly associated with chronological age, with GDF15 (*r* = 0.39), activin A (*r* = 0.30), and TNFR1 (*r* = 0.26) having the strongest relationships (all *p* < 0.01) (Supplemental Table 3). Men had higher levels of activin A, Fas, GDF15, ICAM1, MMP9, PARC, SOST, TNFα, TNFR1, and TNFR2 than women, whereas women had higher levels of MDC, RAGE, RANTES, and uPAR than men (Supplemental Table 4).

### 2.3 Senescence biomarkers and risk of death

**FIGURE 1:**
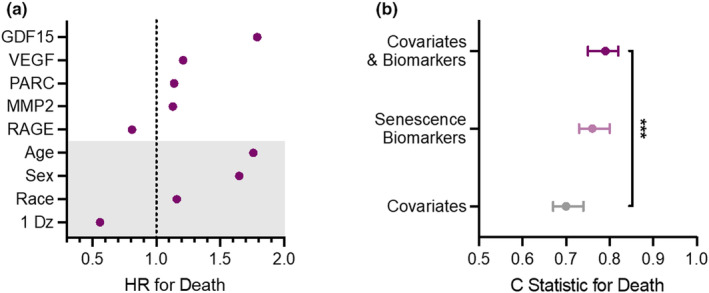
Circulating biomarkers of cellular senescence predict mortality beyond traditional risk factors. (a) The five senescence biomarkers with the strongest associations with mortality from LASSO regression models that include age, sex, race, and presence of a chronic disease at baseline (grey shaded portion of the plot). (b) The C‐statistics indicating the ability of covariates alone, senescence biomarkers alone, and covariates plus biomarkers to predict death. *** indicates a *p* value <0.001 for the change in C‐statistic between the covariates model alone and the model that includes both covariates and biomarkers (training data).

The plasma levels of 14 senescence biomarkers were associated with increased risk of death after adjusting for age, sex, race, and the presence of a chronic condition using LASSO regression (Supplemental Table 5). The five biomarkers with the strongest associations with mortality are displayed in Figure 1a. GDF15 levels were most strongly associated with risk of death (hazard ratio [HR] = 1.79). Plasma concentrations of VEGFA, PARC, and MMP2 conferred the next highest HRs for death amongst the senescence biomarkers (VEGFA HR: 1.21; PARC HR: 1.14; MMP2: HR 1.13). By contrast, lower levels of RAGE were associated with an increased risk of death (HR: 0.81).

In an initial model including only age, sex, race, and presence of a chronic condition, the c‐statistic for the risk of death during follow up was 0.70 (95% CI: 0.67, 0.74) in the training dataset, which represented a random sample of 80% of the study population (Figure 1b; Supplemental Table 5). The addition of senescence biomarkers to the initial model significantly improved the prediction of death to 0.79 (95% CI: 0.76, 0.82) (Figure 1b; Supplemental Table 5; *p* value<0.001). The same biomarkers were also studied in the test dataset (the remaining 20% of the study population); however, the difference in c‐statistics between the covariate model (0.68, 95% CI: 0.60, 0.75) and covariate plus biomarker model (0.72, 95% CI: 0.66, 0.79) was not statistically significant (*p* value = 0.13). Of note, the c‐statistic for the senescence biomarkers alone was 0.76 (95% CI: 0.73, 0.80) in the training data set and 0.70 (95% CI: 0.63, 0.77) in the testing data set.

LASSO models do not provide p values or confidence intervals, making it more difficult to interpret statistical significance. Therefore, we also studied the variables selected by the LASSO models (Supplemental Table 5) in Cox proportional hazards regression models to provide an estimate of the variability of the hazard ratios. Higher levels of GDF15, VEGFA, PARC, and MMP2, and lower levels of RAGE, were significantly associated with an increased risk of mortality in the overall population (Supplemental Table 6; all *p* values <0.005). We also studied the performance of models that included GDF15 alone, and GDF15 plus RAGE (Supplemental Table 6). We found that model performance changed only slightly when only GDF15 was included compared to the full list of SASP proteins selected by the LASSO model (c statistic = 0.76 for the full model and 0.74 with age, sex, race, number of conditions, and GDF15 alone).

**FIGURE 2:**
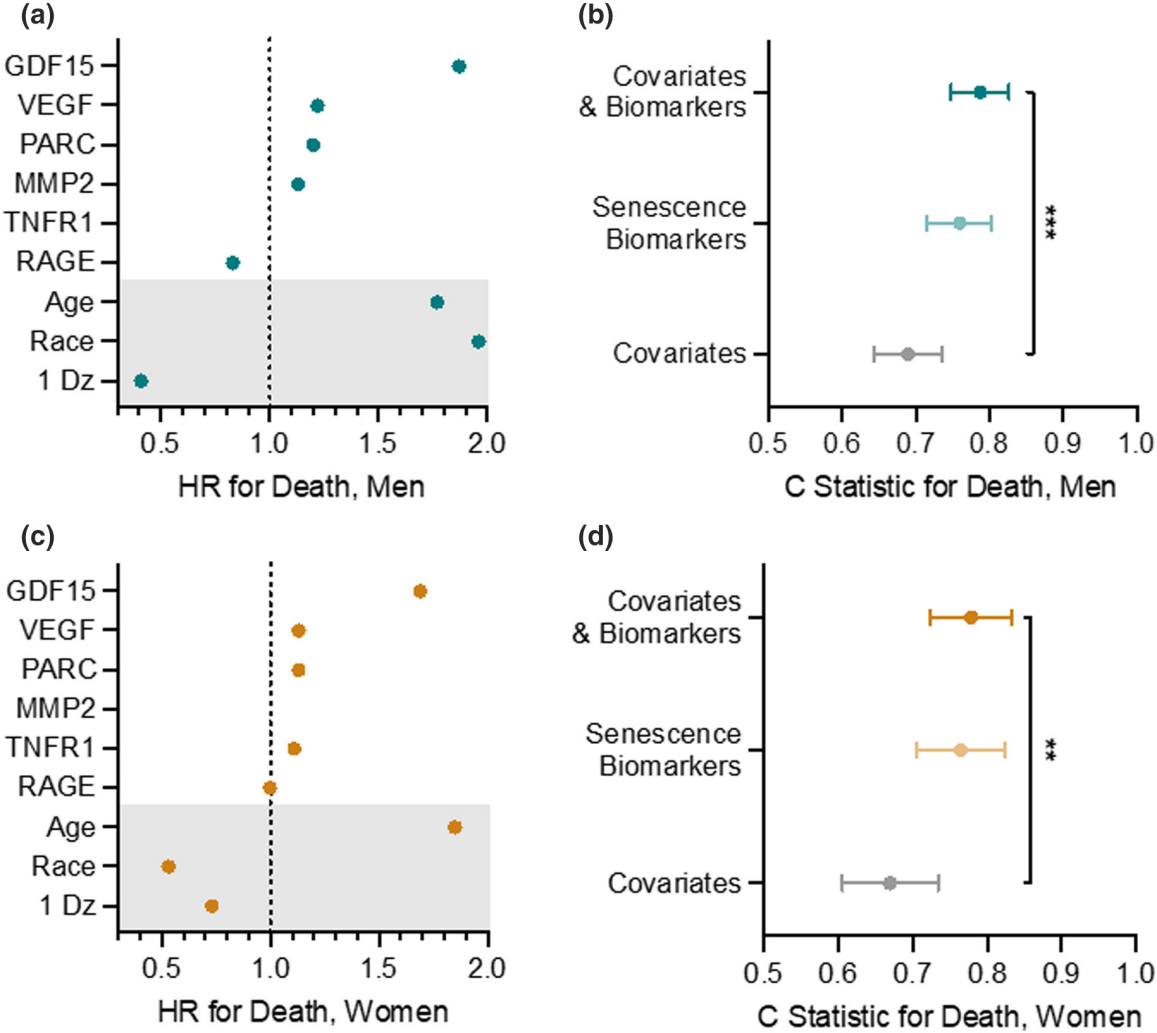
Results of LASSO models run separately for men and women. Hazard ratios are presented for the top five biomarkers that were significant in overall models for men and women. (a) Senescence biomarkers associated with mortality in men in LASSO regression models including age, race, and presence of a chronic disease at baseline. (b) C‐statistics indicating the ability of covariates alone, senescence biomarkers alone, and covariates plus biomarkers to predict death in men. (c) Senescence biomarkers associated with mortality in women in LASSO regression models including age, race, and presence of a chronic disease at baseline. (d) The C‐statistics indicating the ability of covariates alone, senescence biomarkers alone, and covariates plus biomarkers to predict death in women. *** and ** denote *p* < 0.001 and 0.004, respectively.

Finally, we also examined whether model performance differed in men compared to women. In men, GDF15 levels, again followed by VEGFA, RAGE, and PARC, were most strongly associated with mortality even after adjusting for age, race, and the presence of one disease (Figure 2a). As observed in the overall study population, the addition of senescence biomarkers to the covariates significantly improved the performance of the model at predicting mortality from 0.69 (95% CI: 0.64, 0.74) to 0.79 (95% CI: 0.75, 0.83) in men within the training data set (p < 0.001; Figure 2b). Similarly, GDF15 levels, followed by VEGFA, PARC, and TNFR1 but not RAGE, were most strongly associated with mortality in women, even after adjusting for baseline covariates (Figure 2c). Again, the addition of senescence biomarkers to covariates in the model increased the prediction of mortality by covariates alone from 0.67 (95% CI: 0.61, 0.73) to 0.78 (95% CI: 0.72, 0.83) in women within the training data set (*p* = 0.004; Figure 2d).

## 3 DISCUSSION

There is compelling evidence for cellular senescence as a fundamental mechanism of aging. It is therefore plausible that biomarkers of senescent cell burden may be prognostic for mortality. In older adults with zero or only one chronic condition at baseline, we found that candidate biomarkers of cellular senescence predicted mortality beyond traditional demographic risk factors, including chronological age, sex, and the presence of a chronic disease. In particular, higher levels of GDF15, VEGFA, PARC, and MMP2, and lower levels of RAGE, were significantly associated with an increased risk of death. These results suggest that blood‐based biomarkers of cellular senescence in older adults may be informative predictors of biological age and clinically important health outcomes, including death.

(Fielding et al., 2022; Schafer et al., 2020) As expected, chronologic age and male sex were strong predictors of mortality. However, 14 senescence‐related proteins contributed to an increased predictive capacity for mortality beyond the traditional risk factors. In particular, GDF15 was most strongly associated with mortality in the overall models, even after accounting for demographic characteristics. In addition, inclusion of only GDF15 resulted in similar model performance to models that included 14 SASP proteins. These data suggest that GDF15 is driving much of the predictive capacity of the SASP markers studied. GDF15 is a stress‐induced cytokine, and is upregulated by several inflammatory or stress‐regulated proteins. (Wischhusen et al., 2020) Higher GDF15 levels have been observed in older persons, persons with heart disease, and in persons with specific cancers. (Wischhusen et al., 2020) Our results are in agreement with previous studies showing that elevated levels of GDF15 are associated with an increased risk of death in populations of all ages, even after adjustment for several risk factors (Eggers et al., 2013; Ho et al., 2018; Rohatgi et al., 2012).

After GDF15, lower RAGE levels were most strongly associated with mortality. RAGE binds advanced glycation end products to activate pro‐inflammatory responses, and higher levels have been observed in persons with several chronic conditions. (Bierhaus et al., 2005) However, prior data regarding associations between RAGE and mortality are mixed (Erusalimsky, 2021). Some studies suggest that higher levels of RAGE are associated with an increased risk of mortality, particularly in persons with diabetes or heart disease (Erusalimsky, 2021; Ho et al., 2018). Higher RAGE levels have also been associated with an increased risk of mortality in frail, but not non‐frail older adults (Butcher et al., 2019). By contrast, lower levels have been associated with mortality in persons free from cardiovascular disease (Grauen Larsen et al., 2019; Selvin et al., 2013). The explanation for these inconsistent results is unclear. Basta and colleagues have found that persons with lower levels of RAGE had a higher systemic inflammatory state. (Basta et al., 2006) Additionally, mouse models have shown associations with anti‐inflammatory and vascularprotective effects of RAGE. (Bucciarelli et al., 2002; Park et al., 1998) Therefore, previous inconsistent findings might be explained if higher RAGE levels confer a level of protection in healthier adults, but not in adults with established chronic diseases, where other factors may oppose the protective effect. Our study population was largely free of chronic disease at baseline, and our results are similar to studies of persons without cardiovascular diseases. (Butcher et al., 2019; Grauen Larsen et al., 2019; Selvin et al., 2013).

VEGFA had the third strongest association with mortality. Data regarding associations between VEGFA levels and mortality in general populations are limited. VEGFA is upregulated by cardiomyocytes during inflammation, mechanical stress, and cytokine stimulation, and higher levels have been associated with a range of cardiovascular disease and poor cardiovascular outcomes. (Braile et al., 2020) Similarly, VEGFA induces angiogenesis and higher levels of VEGFA are associated with tumor survival, growth, and metastasis. (Saman et al., 2020) The role of VEGFA in predicting mortality in a relatively healthy aging population is currently unclear; however, the role of VEGFA in both cardiovascular disease and cancer progression make it a plausible biomarker for predicting mortality due to these conditions. Our data suggest that VEGFA may add to predictive models for mortality in combination with demographic characteristics, number of chronic conditions, GDF15, and RAGE levels.

We also found that higher levels of PARC and MMP2 had the next strongest associations with mortality. Higher levels of PARC (also known as CCL18) have been previously associated with an increased risk of death in patients with a range of chronic diseases, including chronic obstructive pulmonary disease, (Sin et al., 2011) idiopathic pulmonary fibrosis, (Prasse et al., 2009) coronary artery disease, (de Jager et al., 2012) and cancer progression. (Cardoso et al., 2021) Similarly, elevated MMP2 levels have been previously associated with an increased risk of death in patients with acute coronary syndrome (Dhillon et al., 2009) and several cancers. (Jiang & Li, 2021; Wen et al., 2014) Our results suggest that higher levels of PARC and MMP2 may also predict death even in relatively healthy older adults.

Finally, we found that the strongest predictors of mortality in both men and women were GDF15, VEGFA, and PARC levels. Similarly, RAGE was selected as a contributor to mortality prediction in LASSO models for both men and women, but the hazard ratio for women was 0.997, suggesting only a minor contribution in women. These results are consistent with previous findings in colorectal cancer, where lower levels of soluble RAGE were associated with colorectal cancer in men, but no association was observed in women. (Aglago et al., 2021) In addition, MMP2 levels were associated with mortality in men, but not women, while TNFR1 levels were associated with mortality in women, but not men. We also found that non‐white race was a risk factor for mortality in men, but a protective factor in women. Unfortunately, sample sizes for these models are small, considering the number of potential predictor variables. In particular, it was not possible to further explore the impact of non‐white race separately in men and women because there were only 33 non‐White men and 28 non‐White women in the study sample. Thus, these data should be considered preliminary. However, these data suggest that sex‐specific biomarker models of aging may need to be explored in future studies with large enough samples of both men and women.

Strengths of our study include the cohort study design. We have previously shown that candidate senescence biomarkers are associated with chronologic age and clinical manifestations of advanced biological age in older adults, including frailty index, adverse postsurgical outcomes (e.g., complications and rehospitalization), and limitations in physical function (Fielding et al., 2022; Schafer et al., 2020). However, previous studies have focused on crosssectional associations or condition‐specific outcomes in persons with established disease or geriatric syndromes. It can be difficult in crosssectional studies to determine if elevated biomarker levels preceded the development of the condition or if they were a result of the condition. To prove that these markers are useful for predicting outcomes of aging, it is necessary for these markers to be present prior to the onset of a particular outcome of interest. It is also necessary for these markers to improve the prediction obtained using aging measures that can be more easily collected (e.g., chronologic age and sex). Our cohort study design allowed us to identify persons with zero or only one chronic disease at baseline, and to follow them passively through their medical records until death. This design ensured that the exposure (SASP biomarker measures) preceded the development of our outcome (death), and allowed us to examine whether these biomarkers are useful predictors of mortality.

A second strength of our study was the use of penalized regression (LASSO regression) to study associations between participant characteristics, presence of chronic disease, senescence biomarkers, and mortality. LASSO regression is well‐suited for addressing multicollinearity among the predictor variables, and provides a sparse, interpretable model that avoids overfitting. Additionally, we ran Cox proportional hazard models to provide a sense of the variability in the hazard ratios. We note, however, that the Cox models may be overfit, and thus, the LASSO results should be considered as the primary findings. Additionally, although we had a large sample size, efforts to validate our findings in the training data set were challenged by the relatively small sample size of the testing data set, representative of 20% of the study population. Similar trends were observed, but statistical power was more limited, particularly in sex‐specific analyses.

Other limitations of this study include the relatively homogenous study population drawn from a single midwest region. In particular, the population was predominantly white and non‐Hispanic. It is therefore necessary to repeat these analyses in populations of other races, ethnicities, and residing in other areas of the country to determine whether the results are broadly generalizable.

Surprisingly, we found that persons with one condition at baseline were at a reduced risk of mortality compared to persons without one condition. Age did not differ significantly between persons with and without a condition reported in the medical record (median age of 71.4 years; IQR: 67.3, 75.4) for those with 0 conditions and 71.1 years (IQR: 67.4, 74.8) for those with 1 condition at baseline; (*p* = 0.40). Therefore, differences in age did not account for mortality differences observed between the two groups. It is possible that some of the persons we identified as having no conditions at baseline may have had undiagnosed conditions that were not recognized at the time the SASP biomarkers were measured. Assuming that such conditions placed these persons at higher risk for mortality, failing to account for these conditions would most likely bias our results toward no association; thus, our results may be conservative. However, it is also possible that persons with a condition may have been treated for their condition, and these results may reflect beneficial effects of treatments or therapies for the diagnosed conditions. Arthritis, hyperlipidemia, and cancers were the most common conditions in this population. In a sensitivity analysis, we found that persons with arthritis were not at a lower risk of death, but persons with hyperlipidemia were at a lower risk of death during follow‐up compared to persons without these conditions (arthritis HR: 0.84, 95% CI:0.56, 1.27; hyperlipidemia HR: 0.26 [0.13, 0.53]). Hyperlipidemia is routinely treated with statins, and statins have been associated with a decreased risk of all‐cause mortality in observational cohort studies. (Baigent et al., 2005) Unfortunately, prescription medication information was not available for this population, and further studies are necessary to assess associations between such treatments and SASP levels. Cancer was the third most common condition in this population; however, the ICD codes used to identify cancers for this cohort are broad, and include some less severe diagnoses such as carcinomas in situ (*n* = 9). In addition, the number of persons with any single cancer was very small in this dataset (most common diagnoses were breast cancer [*n* = 21] and prostate cancer [*n* = 18]). The limited number of persons with any single cancer made it impossible to explore associations between cancer types and mortality. We note, however, that adjusting for having any condition at baseline did not significantly change the contribution of SASP biomarkers to prediction of mortality. These results suggest that having any of these conditions did not confound the association between SASP biomarkers and mortality.

We note that our study population was healthier than the average general population 60 years of age or older. We have previously reported that 77% of persons 65 years and older in this geographic region have 2 or more chronic conditions. (Rocca et al., 2014) Therefore, our results may not apply to the average person 60 years or older. We hypothesize that higher levels of GDF15, VEGFA, PARC, and MMP2, and lower levels of RAGE, will also predict mortality in persons with more than one chronic condition; however, further studies are necessary to test this hypothesis. In addition, we hypothesize that younger persons with evidence of accelerated aging (e.g., a high burden of chronic disease in early or midlife) will also show changes in these biomarkers. Further studies are therefore necessary in younger populations to determine whether these biomarkers may be useful in identifying persons who are at high risk of accelerated aging.

In summary, biomarkers of cellular senescence improved prediction of mortality beyond traditional demographic and clinical characteristics, suggesting that circulating concentrations of these proteins may reflect a fundamental mechanism of aging that ultimately leads to death.

## 4 METHODS

This study was reviewed and approved by the Mayo Clinic and Olmsted Medical Center Institutional Review Boards (Mayo Clinic: #18–006044; Olmsted Medical Center: #035‐OMC‐18).

### 4.1 Study population

Study participants were selected from persons 65 years of age or older participating in the Mayo Clinic Biobank (Olson et al., 2019). Briefly, the Mayo Clinic Biobank is an institutional resource including volunteers who have donated biological specimens, provided risk factor data, and have given permission to access clinical data from their EHR for clinical research studies. Participants were contacted as part of a pre‐scheduled medical examination at Mayo Clinic between April 2009 and September 2015. All participants were 18 years or older at the time of enrollment. Approximately 57,000 participants have been included, and 24,224 of these participants were 65 years of age or older at the time of enrollment and had available plasma specimens.

We used the resources of the Rochester Epidemiology Project (REP) medical records‐linkage system to identify persons who had none or only one chronic disease at the time of the blood draw. We considered 20 chronic conditions defined by the Department of Health and Human Services as important for studies of multimorbidity in aging (Supplemental Table 1) (Goodman et al., 2013). The REP has been previously described (Rocca et al., 2018). Briefly, this system links and archives billing codes generated by the participating healthcare institutions at health care visits (inpatient, outpatient, emergency room, or other) for persons living in a 27‐county region of south‐eastern Minnesota and west‐central Wisconsin (Rocca et al., 2018). A random sample of 2000 persons who had codes for zero or only one chronic condition in the previous 5 years was selected for this study.

### 4.2 Senescence biomarkers (exposures)

Proteins were selected based on previous work (Schafer et al., 2020), and full names and abbreviations are shown in Supplemental Table 2. The concentrations of target proteins were quantified using commercially available multiplex magnetic bead‐based immunoassays (R&D Systems) on the Luminex xMAP multianalyte profiling platform and analyzed on MAGPIX System (Merck Millipore) using 220 μL of plasma. MMP9, MMP2, PARC, RANTES, PAI1 and MPO were analyzed on a standard 6‐plex panel using 20 μL plasma diluted 1:100. MMP1, ICAM1, GDF15, TNFRI, uPAR, and Fas were run on a standard 8‐plex panel using 30 μL plasma diluted 1:2. MMP7, MDC, RAGE, SOST, OPN, and TNFR2 were assessed as part of a standard 8‐plex panel using 30 μL plasma diluted 1:2. Eotaxin, MCP1, PDGF‐AA, PDGF‐AB, and VEGF were assessed as part of a performance 12‐plex assay using 30 μL plasma diluted 1:2. TNFα and IL7 were run on a high‐sensitivity 4‐plex panel using 55 μl plasma diluted 1:2 (100ul assay volume). Activin A concentration was determined by a Quantikine ELISA Kit (R&D Systems) using 55 μl plasma diluted 1:2. All assays were performed according to the manufacturer's protocols. Assay performance characteristics are reported in Supplemental Table 7, and a matrix showing the correlations between the SASP biomarkers is presented in Supplemental Table 8.

### 4.3 Mortality (outcome)

We followed persons in the study through the REP to identify persons who died during follow‐up. The REP includes dates of death from all health care providers that participate in the REP, from Minnesota State death certificates, and from the National Death Index (NDI+) for persons who died outside the state of Minnesota. Study subjects were followed from date of plasma collection through last clinical visit date, death, or 04/30/2022 (last available follow‐up information for the cohort), whichever came first.

### 4.4 Data analysis

Patient characteristics were summarized as number and percent. Correlations between pairs of SASP biomarkers and the SASP biomarkers and age were assessed using Spearman correlation coefficients. Differences in SASP biomarker levels between men and women were assessed with linear regression of natural log‐transformed SASP biomarker on sex, adjusted for age and race. Least absolute shrinkage and selection operator (LASSO) regression was used to create predictive models for mortality. (Tibshirani, 1996) LASSO selects predictors by shrinking coefficients for less influential predictors to zero, resulting in a final sparse prediction model. Seventeen people had missing data and were excluded from the LASSO modeling. All SASP biomarkers were log transformed after being evaluated for skewed distributions, and, if appropriate, winsorized. Biomarkers were then standardized by subtracting the mean and dividing by the standard deviation. An 80% random sample was used for the training dataset; the remaining 20% was used for the testing dataset. To avoid overfitting, 10‐fold cross‐validation was used to select the optimal tuning parameter for the training dataset, defined as the minimum of the cross‐validation error curve for the partial likelihood. The resulting model was subsequently applied in the testing dataset, and C‐statistics were calculated to assess model performance in both the training and testing datasets. Models were fit using (1) age, sex, race (white vs non‐white), and number of chronic conditions at baseline (0 vs 1), (2) all SASP biomarkers and (3) all variables (forcing in age, sex, race, and number of conditions). We also ran models separately within men and women.

We note that LASSO models do not provide 95% confidence intervals or P values, so Cox proportional hazards regression models were run with age, sex, race, number of chronic conditions at baseline and the SASP biomarkers selected from the LASSO regression as predictors. These results indicated that GDF15, RAGE, VEGFA, PARC, and MMP2 were most strongly associated with an increased risk of death (Supplemental Table 5). HR estimates are slightly different between the LASSO and Cox models. However, because LASSO models better account for correlation between the variables, we present LASSO hazard ratios in the primary results. Analyses were conducted using SAS statistical software, version 9.4 (SAS Institute Inc, Cary, NC) and R version 4.0.3.

## AUTHOR CONTRIBUTIONS

Conception or design of the work: J.L.S., J.E.O., and N.K.L.; Acquisition, analysis, or interpretation of data for the work: J.L.S., S.A.W., E.J.A., M.E.M., T.A.W., A.A.H., J.E.O., W.A.R., S.J.B., and N.K.L.; Drafting of the work: J.L.S., S.A.W., and N.K.L.; Critical revision: J.L.S., S.A.W., E.J.A., M.E.M., M.M.M., T.A.W., A.A.H., J.E.O., W.A.R., A.K.P., S.R.C., R.A.F., S.J.B., and N.K.L.; Final approval of the version to be published: J.L.S., S.A.W., E.J.A., M.E.M., M.M.M., T.A.W., A.A.H., J.E.O., W.A.R., A.K.P., S.R.C., R.A.F., S.J.B., and N.K.L.

## FUNDING INFORMATION

This research was funded by the National Institutes of Health, the National Institute on Aging R56 AG60907 (S.R.C., N.K.L.), R01 AG55529 (M.M., S.R.C., N.K.L.), and the Glenn Foundation for Medical Research (N.K.L.). This study used the resources of the Rochester Epidemiology Project (REP) medical records‐linkage system, which is supported by the National Institute on Aging (NIA; R33 AG 058738), by the Mayo Clinic Research Committee, and by fees paid annually by REP users. The content of this article is solely the responsibility of the authors and does not represent the official views of the National Institutes of Health (NIH) or the Mayo Clinic. In addition, R.A.F. is partially supported by the US Department of Agriculture (USDA), under agreement No. 58–8050–9‐004, by NIH Boston Claude D. Pepper Center (OAIC; 1P30AG031679). Any opinions, findings, conclusions, or recommendations expressed in this publication are those of the authors and do not necessarily reflect the view of the USDA.

## CONFLICT OF INTEREST STATEMENT

N.K.L. and Mayo Clinic have patent applications related to this research licensed to or filed by commercial entities. The research reported here has been reviewed by the Mayo Clinic Conflict of Interest Review Board and is being conducted in compliance with Mayo Clinic Conflict of Interest policies.

## Supporting information


**Data S1.** Supporting Information.

## Data Availability

Data used for this manuscript are available on request to Dr. St. Sauver and Dr. LeBrasseur. Datasets contain identifiable patient information. Therefore, limited datasets may be shared with enactment of Data Use Agreements to ensure compliance with Health Insurance Portability and Accountability (HIPAA) requirements.

## References

[acel14006-bib-0001] Aglago, E. K. , Rinaldi, S. , Freisling, H. , Jiao, L. , Hughes, D. J. , Fedirko, V. , Schalkwijk, C. G. , Weiderpass, E. , Dahm, C. C. , Overvad, K. , Eriksen, A. K. , Kyrø, C. , Boutron‐Ruault, M. C. , Rothwell, J. A. , Severi, G. , Katzke, V. , Kühn, T. , Schulze, M. B. , Aleksandrova, K. , & Jenab, M. (2021). Soluble receptor for advanced glycation end‐products (sRAGE) and colorectal cancer risk: A case‐control study nested within a European prospective cohort. Cancer Epidemiology, Biomarkers & Prevention, 30(1), 182–192. 10.1158/1055-9965.EPI-20-0855 33082206

[acel14006-bib-0002] Aversa, Z. , Atkinson, E. J. , Carmona, E. M. , White, T. A. , Heeren, A. A. , Jachim, S. K. , Zhang, X. , Cummings, S. R. , Chiarella, S. E. , Limper, A. H. , & LeBrasseur, N. K. (2023). Biomarkers of cellular senescence in idiopathic pulmonary fibrosis. Respiratory Research, 24(1), 101. 10.1186/s12931-023-02403-8 37029417 PMC10080755

[acel14006-bib-0003] Baigent, C. , Keech, A. , Kearney, P. M. , Blackwell, L. , Buck, G. , Pollicino, C. , Kirby, A. , Sourjina, T. , Peto, R. , Collins, R. , Simes, R. , & Cholesterol Treatment Trialists, C . (2005). Efficacy and safety of cholesterol‐lowering treatment: Prospective meta‐analysis of data from 90,056 participants in 14 randomised trials of statins. Lancet, 366(9493), 1267–1278. 10.1016/S0140-6736(05)67394-1 16214597

[acel14006-bib-0004] Barnett, K. , Mercer, S. W. , Norbury, M. , Watt, G. , Wyke, S. , & Guthrie, B. (2012). Epidemiology of multimorbidity and implications for health care, research, and medical education: A crosssectional study. Lancet, 380(9836), 37–43. 10.1016/S0140-6736(12)60240-2 22579043

[acel14006-bib-0005] Basta, G. , Sironi, A. M. , Lazzerini, G. , Del Turco, S. , Buzzigoli, E. , Casolaro, A. , Natali, A. , Ferrannini, E. , & Gastaldelli, A. (2006). Circulating soluble receptor for advanced glycation end products is inversely associated with glycemic control and S100A12 protein. The Journal of Clinical Endocrinology and Metabolism, 91(11), 4628–4634. 10.1210/jc.2005-2559 16926247

[acel14006-bib-0006] Bierhaus, A. , Humpert, P. M. , Morcos, M. , Wendt, T. , Chavakis, T. , Arnold, B. , Stern, D. M. , & Nawroth, P. P. (2005). Understanding RAGE, the receptor for advanced glycation end products. Journal of Molecular Medicine (Berlin, Germany), 83(11), 876–886. 10.1007/s00109-005-0688-7 16133426

[acel14006-bib-0007] Braile, M. , Marcella, S. , Cristinziano, L. , Galdiero, M. R. , Modestino, L. , Ferrara, A. L. , Varricchi, G. , Marone, G. , & Loffredo, S. (2020). VEGF‐A in Cardiomyocytes and heart diseases. International Journal of Molecular Sciences, 21(15), 1–18. 10.3390/ijms21155294 PMC7432634 32722551

[acel14006-bib-0008] Bucciarelli, L. G. , Wendt, T. , Qu, W. , Lu, Y. , Lalla, E. , Rong, L. L. , Goova, M. T. , Moser, B. , Kislinger, T. , Lee, D. C. , Kashyap, Y. , Stern, D. M. , & Schmidt, A. M. (2002). RAGE blockade stabilizes established atherosclerosis in diabetic apolipoprotein E‐null mice. Circulation, 106(22), 2827–2835. 10.1161/01.cir.0000039325.03698.36 12451010

[acel14006-bib-0009] Butcher, L. , Carnicero, J. A. , Gomez Cabrero, D. , Dartigues, J. F. , Peres, K. , Garcia‐Garcia, F. J. , Rodriguez‐Mañas, L. , Erusalimsky, J. D. , & Consortium, F . (2019). Increased levels of soluble receptor for advanced glycation end‐products (RAGE) are associated with a higher risk of mortality in frail older adults. Age and Ageing, 48(5), 696–702. 10.1093/ageing/afz073 31211360

[acel14006-bib-0010] Cardoso, A. P. , Pinto, M. L. , Castro, F. , Costa, A. M. , Marques‐Magalhaes, A. , Canha‐Borges, A. , Cruz, T. , Velho, S. , & Oliveira, M. J. (2021). The immunosuppressive and pro‐tumor functions of CCL18 at the tumor microenvironment. Cytokine & Growth Factor Reviews, 60, 107–119. 10.1016/j.cytogfr.2021.03.005 33863622

[acel14006-bib-0011] Choudhery, M. S. , Badowski, M. , Muise, A. , Pierce, J. , & Harris, D. T. (2014). Donor age negatively impacts adipose tissue‐derived mesenchymal stem cell expansion and differentiation. Journal of Translational Medicine, 12, 1–14. 10.1186/1479-5876-12-8 24397850 PMC3895760

[acel14006-bib-0012] Coppe, J. P. , Patil, C. K. , Rodier, F. , Krtolica, A. , Beausejour, C. M. , Parrinello, S. , Hodgson, J. G. , Chin, K. , Desprez, P. Y. , & Campisi, J. (2010). A human‐like senescence‐associated secretory phenotype is conserved in mouse cells dependent on physiological oxygen. PLoS One, 5(2), e9188. 10.1371/journal.pone.0009188 20169192 PMC2820538

[acel14006-bib-0013] de Jager, S. C. , Bongaerts, B. W. , Weber, M. , Kraaijeveld, A. O. , Rousch, M. , Dimmeler, S. , van Dieijen‐Visser, M. P. , Cleutjens, K. B. , Nelemans, P. J. , van Berkel, T. J. , & Biessen, E. A. (2012). Chemokines CCL3/MIP1alpha, CCL5/RANTES and CCL18/PARC are independent risk predictors of short‐term mortality in patients with acute coronary syndromes. PLoS One, 7(9), e45804. 10.1371/journal.pone.0045804 23029252 PMC3448678

[acel14006-bib-0014] Dhillon, O. S. , Khan, S. Q. , Narayan, H. K. , Ng, K. H. , Mohammed, N. , Quinn, P. A. , Squire IB , Davies JE . &. Ng, L. L. (2009). Matrix metalloproteinase‐2 predicts mortality in patients with acute coronary syndrome. Clinical Science (London, England), 118(4), 249–257. doi:10.1042/CS20090226 19583569

[acel14006-bib-0015] Eggers, K. M. , Kempf, T. , Wallentin, L. , Wollert, K. C. , & Lind, L. (2013). Change in growth differentiation factor 15 concentrations over time independently predicts mortality in community‐dwelling elderly individuals. Clinical Chemistry, 59(7), 1091–1098. 10.1373/clinchem.2012.201210 23529704

[acel14006-bib-0016] Englund, D. A. , Jolliffe, A. , Aversa, Z. , Zhang, X. , Sturmlechner, I. , Sakamoto, A. E. , Zeidler, J. D. , Warner, G. M. , McNnch, C. , White, T. A. , Chini, E. N. , Baker, D. J. , van Deursen, J. M. , & LeBrasseur, N. K. (2023). p21 induces a senescence program and skeletal muscle dysfunction. Molecular Metabolism, 67, 101652. 10.1016/j.molmet.2022.101652 36509362 PMC9800630

[acel14006-bib-0017] Erusalimsky, J. D. (2021). The use of the soluble receptor for advanced glycation‐end products (sRAGE) as a potential biomarker of disease risk and adverse outcomes. Redox Biology, 42, 101958. 10.1016/j.redox.2021.101958 33839083 PMC8113049

[acel14006-bib-0018] Farr, J. N. , Fraser, D. G. , Wang, H. , Jaehn, K. , Ogrodnik, M. B. , Weivoda, M. M. , Drake, M. T. , Tchkonia, T. , Nk, L. B. , Kirkland, J. L. , Bonewald, L. F. , Pignolo, R. J. , Monroe, D. G. , & Khosla, S. (2016). Identification of senescent cells in the bone microenvironment. Journal of Bone and Mineral Research, 31(11), 1920–1929. 10.1002/jbmr.2892 27341653 PMC5289710

[acel14006-bib-0019] Fielding, R. A. , Atkinson, E. J. , Aversa, Z. , White, T. A. , Heeren, A. A. , Achenbach, S. J. , Mielke, M. M. , Cummings, S. R. , Pahor, M. , Leeuwenburgh, C. , & LeBrasseur, N. K. (2022). Associations between biomarkers of cellular senescence and physical function in humans: Observations from the lifestyle interventions for elders (LIFE) study. Geroscience, 44(6), 2757–2770. 10.1007/s11357-022-00685-2 36367600 PMC9768064

[acel14006-bib-0020] Goodman, R. A. , Posner, S. F. , Huang, E. S. , Parekh, A. K. , & Koh, H. K. (2013). Defining and measuring chronic conditions: Imperatives for research, policy, program, and practice. Prev Chronic Disease, 10, E66. 10.5888/pcd10.120239 PMC3652713 23618546

[acel14006-bib-0021] Grauen Larsen, H. , Marinkovic, G. , Nilsson, P. M. , Nilsson, J. , Engstrom, G. , Melander, O. , Orho‐Melander, M. , & Schiopu, A. (2019). High plasma sRAGE (soluble receptor for advanced glycation end products) is associated with slower carotid intima‐media thickness progression and lower risk for first‐time coronary events and mortality. Arteriosclerosis, Thrombosis, and Vascular Biology, 39(5), 925–933. 10.1161/ATVBAHA.118.312319 30917679

[acel14006-bib-0022] Ho, J. E. , Lyass, A. , Courchesne, P. , Chen, G. , Liu, C. , Yin, X. , Hwang, S. J. , Massaro, J. M. , Larson, M. G. , & Levy, D. (2018). Protein biomarkers of cardiovascular disease and mortality in the community. Journal of the American Heart Association, 7(14), 1–11. 10.1161/JAHA.117.008108 PMC6064847 30006491

[acel14006-bib-0023] Jiang, H. , & Li, H. (2021). Prognostic values of tumoral MMP2 and MMP9 overexpression in breast cancer: A systematic review and meta‐analysis. BMC Cancer, 21(1), 149. 10.1186/s12885-021-07860-2 33568081 PMC7877076

[acel14006-bib-0024] LeBrasseur, N. K. , Tchkonia, T. , & Kirkland, J. L. (2015). Cellular senescence and the biology of aging, disease, and frailty. Nestle Nutrition Institute Workshop Series, 83, 11–18. 10.1159/000382054 26485647 PMC4780350

[acel14006-bib-0025] Lopes‐Paciencia, S. , Saint‐Germain, E. , Rowell, M. C. , Ruiz, A. F. , Kalegari, P. , & Ferbeyre, G. (2019). The senescence‐associated secretory phenotype and its regulation. Cytokine, 117, 15–22. 10.1016/j.cyto.2019.01.013 30776684

[acel14006-bib-0026] Neumann, P. , Lenz, D. E. , Streit, W. J. , & Bechmann, I. (2023). Is microglial dystrophy a form of cellular senescence? An analysis of senescence markers in the aged human brain. Glia, 71(2), 377–390. 10.1002/glia.24282 36286188

[acel14006-bib-0027] Newgard, C. B. , & Sharpless, N. E. (2013). Coming of age: Molecular drivers of aging and therapeutic opportunities. The Journal of Clinical Investigation, 123(3), 946–950. 10.1172/JCI68833 23454756 PMC3582156

[acel14006-bib-0028] Olson, J. E. , Ryu, E. , Hathcock, M. A. , Gupta, R. , Bublitz, J. T. , Takahashi, P. Y. , Bielinski, S. J. , St Sauver, J. L. , Meagher, K. , Sharp, R. R. , Thibodeau, S. N. , Cicek, M. , & Cerhan, J. R. (2019). Characteristics and utilisation of the Mayo Clinic biobank, a clinic‐based prospective collection in the USA: Cohort profile. BMJ Open, 9(11), e032707. 10.1136/bmjopen-2019-032707 PMC6858142 31699749

[acel14006-bib-0029] Park, L. , Raman, K. G. , Lee, K. J. , Lu, Y. , Ferran, L. J., Jr. , Chow, W. S. , Stern, D. , & Schmidt, A. M. (1998). Suppression of accelerated diabetic atherosclerosis by the soluble receptor for advanced glycation endproducts. Nature Medicine, 4(9), 1025–1031. 10.1038/2012 9734395

[acel14006-bib-0030] Prasse, A. , Probst, C. , Bargagli, E. , Zissel, G. , Toews, G. B. , Flaherty, K. R. , Oischewski, M. , Rottoli, P. , & Muller‐Quernheim, J. (2009). Serum CC‐chemokine ligand 18 concentration predicts outcome in idiopathic pulmonary fibrosis. American Journal of Respiratory and Critical Care Medicine, 179(8), 717–723. 10.1164/rccm.200808-1201OC 19179488

[acel14006-bib-0031] Rocca, W. A. , Boyd, C. M. , Grossardt, B. R. , Bobo, W. V. , Finney Rutten, L. J. , Roger, V. L. , Ebbert, J. O. , Therneau, T. M. , Yawn, B. P. , & St Sauver, J. L. (2014). Prevalence of multimorbidity in a geographically defined American population: Patterns by age, sex, and race/ethnicity. Mayo Clinic Proceedings, 89(10), 1336–1349. 10.1016/j.mayocp.2014.07.010 25220409 PMC4186914

[acel14006-bib-0032] Rocca, W. A. , Grossardt, B. R. , Brue, S. M. , Bock‐Goodner, C. M. , Chamberlain, A. M. , Wilson, P. M. , Finney Rutten, L. J. , & St Sauver, J. L. (2018). Data resource profile: Expansion of the Rochester epidemiology project medical records‐linkage system (E‐REP). International Journal of Epidemiology, 47(2), 368–368j. 10.1093/ije/dyx268 29346555 PMC5913632

[acel14006-bib-0033] Rohatgi, A. , Patel, P. , Das, S. R. , Ayers, C. R. , Khera, A. , Martinez‐Rumayor, A. , Berry, J. D. , Dk, M. G. , & de Lemos, J. A. (2012). Association of growth differentiation factor‐15 with coronary atherosclerosis and mortality in a young, multiethnic population: Observations from the Dallas heart study. Clinical Chemistry, 58(1), 172–182. 10.1373/clinchem.2011.171926 22065155 PMC3926660

[acel14006-bib-0034] Saman, H. , Raza, S. S. , Uddin, S. , & Rasul, K. (2020). Inducing angiogenesis, a key step in cancer vascularization, and treatment approaches. Cancers (Basel), 12(5), 1–18. 10.3390/cancers12051172 PMC7281705 32384792

[acel14006-bib-0035] Saul, D. , Kosinsky, R. L. , Atkinson, E. J. , Doolittle, M. L. , Zhang, X. , LeBrasseur, N. K. , Pignolo, R. J. , Robbins, P. D. , Niedernhofer, L. J. , Ikeno, Y. , Jurk, D. , Passos, J. F. , Hickson, L. J. , Xue, A. , Monroe, D. G. , Tchkonia, T. , Kirkland, J. L. , Farr, J. N. , & Khosla, S. (2022). A new gene set identifies senescent cells and predicts senescence‐associated pathways across tissues. Nature Communications, 13(1), 4827. 10.1038/s41467-022-32552-1 PMC9381717 35974106

[acel14006-bib-0036] Schafer, M. J. , White, T. A. , Iijima, K. , Haak, A. J. , Ligresti, G. , Atkinson, E. J. , Oberg, A. L. , Birch, J. , Salmonowicz, H. , Zhu, Y. , Mazula, D. L. , Brooks, R. W. , Fuhrmann‐Stroissnigg, H. , Pirtskhalava, T. , Prakash, Y. S. , Tchkonia, T. , Robbins, P. D. , Aubry, M. C. , Passos, J. F. , & LeBrasseur, N. K. (2017). Cellular senescence mediates fibrotic pulmonary disease. Nature Communications, 8, 14532. 10.1038/ncomms14532 PMC5331226 28230051

[acel14006-bib-0037] Schafer, M. J. , Zhang, X. , Kumar, A. , Atkinson, E. J. , Zhu, Y. , Jachim, S. , Mazula, D. L. , Brown, A. K. , Berning, M. , Aversa, Z. , Kotajarvi, B. , Bruce, C. J. , Greason, K. L. , Suri, R. M. , Tracy, R. P. , Cummings, S. R. , White, T. A. , & LeBrasseur, N. K. (2020). The senescence‐associated secretome as an indicator of age and medical risk. JCI Insight, 5(12), 1–13. 10.1172/jci.insight.133668 PMC7406245 32554926

[acel14006-bib-0038] Selvin, E. , Halushka, M. K. , Rawlings, A. M. , Hoogeveen, R. C. , Ballantyne, C. M. , Coresh, J. , & Astor, B. C. (2013). sRAGE and risk of diabetes, cardiovascular disease, and death. Diabetes, 62(6), 2116–2121. 10.2337/db12-1528 23396398 PMC3661610

[acel14006-bib-0039] Sin, D. D. , Miller, B. E. , Duvoix, A. , Man, S. F. , Zhang, X. , Silverman, E. K. , Connett, J. E. , Anthonisen, N. A. , Wise, R. A. , Tashkin, D. , Celli, B. R. , Edwards, L. D. , Locantore, N. , Macnee, W. , Tal‐Singer, R. , Lomas, D. A. , & Investigators, E. (2011). Serum PARC/CCL‐18 concentrations and health outcomes in chronic obstructive pulmonary disease. American Journal of Respiratory and Critical Care Medicine, 183(9), 1187–1192. 10.1164/rccm.201008-1220OC 21216880 PMC3114051

[acel14006-bib-0040] Terlecki‐Zaniewicz, L. , Lämmermann, I. , Latreille, J. , Bobbili, M. R. , Pils, V. , Schosserer, M. , Weinmüllner, R. , Dellago, H. , Skalicky, S. , Pum, D. , Jch, A. , Scheideler, M. , Morizot, F. , Hackl, M. , Gruber, F. , & Grillari, J. (2018). Small extracellular vesicles and their miRNA cargo are anti‐apoptotic members of the senescence‐associated secretory phenotype. Aging (Albany NY), 10(5), 1103–1132. 10.18632/aging.101452 29779019 PMC5990398

[acel14006-bib-0041] Tibshirani, R. (1996). Regression shrinkage and selection via the Lasso. Journal of the Royal Statistical Society Series B‐Methodological, 58(1), 267–288. 10.1111/j.2517-6161.1996.tb02080.x

[acel14006-bib-0042] Wen, X. , Liu, H. , Yu, K. , & Liu, Y. (2014). Matrix metalloproteinase 2 expression and survival of patients with osteosarcoma: A meta‐analysis. Tumour Biology, 35(1), 845–848. 10.1007/s13277-013-1116-1 24037915

[acel14006-bib-0043] Wischhusen, J. , Melero, I. , & Fridman, W. H. (2020). Growth/differentiation Factor‐15 (GDF‐15): From biomarker to novel targetable immune checkpoint. Frontiers in Immunology, 11, 951. 10.3389/fimmu.2020.00951 32508832 PMC7248355

[acel14006-bib-0044] Yousefzadeh, M. J. , Zhao, J. , Bukata, C. , Wade, E. A. , McGowan, S. J. , Angelini, L. A. , Bank, M. P. , Gurkar, A. U. , Ca, M. G. , Calubag, M. F. , Kato, J. I. , Burd, C. E. , Robbins, P. D. , & Niedernhofer, L. J. (2020). Tissue specificity of senescent cell accumulation during physiologic and accelerated aging of mice. Aging Cell, 19(3), e13094. 10.1111/acel.13094 31981461 PMC7059165

