# Supplementary material for: Biomarkers of cellular senescence and risk of death in humans
Source: Aging Cell. 2023 Oct 6;22(12):e14006. doi: 10.1111/acel.14006 (PMC10726868; doi:10.1111/acel.14006)
Supplement: Supplementary file 1 — Data S1. Supporting Information. [file ACEL-22-e14006-s001.docx]

**SUPPORTING INFORMATION**

**Supplemental Table 1.** US Dept. of Health and Human Services – 20 Chronic Conditions and associated International Classification of Diseases (ICD) ICD-9 and ICD-10 codes

| **Condition description** | **CCS** | **CMS (ICD-9 Codes)** | **CMS (ICD-10 Codes)** | **Persons with condition at baseline**  **N (%)** |
| --- | --- | --- | --- | --- |
| Hypertension | 98, 99 | 401.0, 401.1, 401.9, 402.00, 402.01, 402.10,  402.11, 402.90, 402.91, 403.00, 403.01,  403.10, 403.11, 403.90, 403.91, 404.00,  404.01, 404.02, 404.03, 404.10, 404.11,  404.12, 404.13, 404.90, 404.91, 404.92,  404.93, 405.01, 405.09, 405.11, 405.19,  405.91, 405.99, 362.11, 437.2 | H35.031, H35.032, H35.033, H35.039, I10, I11.0, I11.9, I12.0, I12.9, I13.0, I13.10, I13.11, I13.2, I15.0, I15.1, I15.2, I15.8, I15.9, I67.4, N26.2 | 61 (3.2) |
| Congestive heart failure | 108 | 398.91, 402.01, 402.11, 402.91, 404.01,  404.11, 404.91, 404.03, 404.13, 404.93,  428.0, 428.1, 428.20, 428.21, 428.22,  428.23, 428.30, 428.31, 428.32, 428.33,  428.40, 428.41, 428.42, 428.43, 428.9 | I09.81, I11.0, I13.0, I13.2, I50.1, I50.20, I50.21, I50.22, I50.23, I50.30, I50.31, I50.32, I50.33, I50.40, I50.41, I50.42, I50.43, I50.810, I50.811, I50.812, I50.813, I50.814, I50.82, I50.83, I50.84, I50.89, I50.9 | 0 (0) |
| Coronary artery disease | 100, 101 | 410.00, 410.01, 410.02, 410.10, 410.11,  410.12, 410.20, 410.21, 410.22, 410.30,  410.31, 410.32, 410.40, 410.41, 410.42,  410.50, 410.51, 410.52, 410.60, 410.61,  410.62, 410.70, 410.71, 410.72, 410.80,  410.81, 410.82, 410.90, 410.91, 410.92,  411.0, 411.1, 411.81, 411.89, 412, 413.0,  413.1, 413.9, 414.00, 414.01, 414.02,  414.03, 414.04, 414.05, 414.06, 414.07,  414.12, 414.2, 414.3, 414.8, 414.9 | I20.0, I20.1, I20.8, I20.9, I21.01, I21.02, I21.09, I21.11, I21.19, I21.21, I21.29, I21.3,  I21.4, I21.A1, I21.A9, I22.0, I22.1, I22.2, I22.8, I22.9, I23.0, I23.1, I23.2, I23.3, I23.4,  I23.5, I23.6, I23.7, I23.8, I24.0, I24.1, I24.8, I24.9, I25.10, I25.110, I25.111, I25.118, I25.119, I25.2, I25.3, I25.41, I25.42, I25.5, I25.6, I25.700, I25.701, I25.708, I25.709, I25.710, I25.711, I25.718, I25.719, I25.720, I25.721, I25.728, I25.729, I25.730, I25.731, I25.738, I25.739, I25.750, I25.751, I25.758, I25.759, I25.760, I25.761, I25.768, I25.769, I25.790, I25.791, I25.798, I25.799, I25.810, I25.811, I25.812, I25.82, I25.83, I25.84, I25.89, I25.9 | 3 (0.2) |
| Cardiac arrhythmias | 105, 106 | 427.31 | I48.0, I48.1, I48.2, I48.91 | 15 (0.8) |
| Hyperlipidemia | 53 | 272.0, 272.1, 272.2, 272.3, 272.4 | E78.0, E78.1, E78.2, E78.3, E78.4, E78.5 | 108 (5.6) |
| Stroke (including TIAs) | 109–112 | 430, 431, 433.01, 433.11, 433.21, 433.31,  433.81, 433.91, 434.00, 434.01,434.10,  434.11, 434.90, 434.91, 435.0, 435.1, 435.3,  435.8, 435.9, 436, 997.02 | G45.0, G45.1, G45.2, G45.8, G45.9, G46.0, G46.1, G46.2, G46.3, G46.4, G46.5, G46.6, G46.7, G46.8, G97.31, G97.32, I60.00, I60.01, I60.02, I60.10, I60.11, I60.12, I60.20, I60.21, I60.22, I60.30, I60.31, I60.32, I60.4, I60.50, I60.51, I60.52, I60.6, I60.7, I60.8, I60.9, I61.0, I61.1, I61.2, I61.3, I61.4, I61.5, I61.6, I61.8, I61.9, I63.00, I63.02, I63.011, I63.012, I63.013, I63.019, I63.02, I63.031, I63.032, I63.039, I63.09, I63.10, I63.111, I63.112, I63.119, I63.12, I63.131, I63.132, I63.139, I63.19, I63.20, I63.211, I63.212, I63.213, I63.219, I63.22, I63.231, I63.232, I63.233, I63.239, I63.29, I63.30, I63.311, I63.312, I63.313, I63.319, I63.321, I63.322, I63.323, I63.329, I63.331, I63.332, I63.333, I63.339, I63.341, I63.342, I63.343, I63.349, I63.39, I63.40, I63.411, I63.412, I63.413, I63.419, I63.421, I63.422, I63.423, I63.429, I63.431, I63.432, I63.433, I63.439, I63.441, I63.442, I63.443, I63.449, I63.49, I63.50, I63.511, I63.512, I63.513, I63.519, I63.521, I63.522, I63.523, I63.529, I63.531, I63.532, I63.533, I63.539, I63.541, I63.542, I63.543, I63.549, I63.59, I63.6, I63.8, I63.9, I66.01, I66.02, I66.03, I66.09, I66.11, I66.12, I66.13, I66.19, I66.21, I66.22, I66.23, I66.29, I66.3, I66.8, I66.9, I67.841, I67.848, I67.89, I97.810, I97.811, I97.820, I97.821 | 3 (0.2) |
| Arthritis  *(continued on next page)* | 202, 203 | 714.0, 714.1, 714.2, 714.30, 714.31, 714.32,  714.33, 715.00, 715.04, 715.09, 715.10,  715.11, 715.12, 715.13, 715.14, 715.15,  715.16, 715.17, 715.18, 715.20, 715.21,  715.22, 715.23, 715.24, 715.25, 715.26,  715.27, 715.28, 715.30, 715.31, 715.32,  715.33, 715.34, 715.35, 715.36, 715.37,  715.38, 715.80, 715.89, 715.90, 715.91,  715.92, 715.93, 715.94, 715.95, 715.96,  715.97, 715.98, 720.0, 721.0, 721.1, 721.2,  721.3, 721.90, 721.91 | M05.00, M05.011, M05.012, M05.019, M05.021, M05.022, M05.029, M05.031, M05.032, M05.039, M05.041, M05.042, M05.049, M05.051, M05.052, M05.059, M05.061, M05.062, M05.069, M05.071, M05.072, M05.079, M05.09, M05.20, M05.211, M05.212, M05.219, M05.221, M05.222, M05.229, M05.231, M05.232, M05.239, M05.241, M05.242, M05.249, M05.251, M05.252, M05.259, M05.261, M05.262, M05.269, M05.271, M05.272, M05.279, M05.29, M05.30, M05.311, M05.312, M05.319, M05.321, M05.322, M05.329, M05.331, M05.332, M05.339, M05.341, M05.342, M05.349, M05.351, M05.352, M05.359, M05.361, M05.362, M05.369, M05.371, M05.372, M05.379, M05.39, M05.40, M05.411, M05.412, M05.419, M05.421, M05.422, M05.429, M05.431, M05.432, M05.439, M05.441, M05.442, M05.449, M05.451, M05.452, M05.459, M05.461, M05.462, M05.469, M05.471, M05.472, M05.479, M05.49, M05.50, M05.511, M05.512, M05.519, M05.521, M05.522, M05.529, M05.531, M05.532, M05.539, M05.541, M05.542, M05.549, M05.551, M05.552, M05.559, M05.561, M05.562, M05.569, M05.571, M05.572, M05.579, M05.59, M05.60, M05.611, M05.612, M05.619, M05.621, M05.622, M05.629, M05.631, M05.632, M05.639, M05.641, M05.642, M05.649, M05.651, M05.652, M05.659, M05.661, M05.662, M05.669, M05.671, M05.672, M05.679, M05.69, M05.70, M05.711, M05.712, M05.719, M05.721, M05.722, M05.729, M05.731, M05.732, M05.739, M05.741, M05.742, M05.749, M05.751, M05.752, M05.759, M05.761, M05.762, M05.769, M05.771, M05.772, M05.779, M05.79, M05.80, M05.811, M05.812, M05.819, M05.821, M05.822, M05.829, M05.831, M05.832, M05.839, M05.841, M05.842, M05.849, M05.851, M05.852, M05.859, M05.861, M05.862, M05.869, M05.871, M05.872, M05.879, M05.89, M05.9, M06.00, M06.011, M06.012, M06.019, M06.021, M06.022, M06.029, M06.031, M06.032, M06.039, M06.041, M06.042, M06.049, M06.051, M06.052, M06.059, M06.061, M06.062, M06.069, M06.071, M06.072, M06.079, M06.08, M06.09, M06.1, M06.20, M06.211, M06.212, M06.219, M06.221, M06.222, M06.229, M06.231, M06.232, M06.239, M06.241, M06.242, M06.249, M06.251, M06.252, M06.259, M06.261, M06.262, M06.269, M06.271, M06.272, M06.279, M06.28, M06.29, M06.30, M06.311, M06.312, M06.319, M06.321, M06.322, M06.329, M06.331, M06.332, M06.339, M06.341, M06.342, M06.349, M06.351, M06.352, M06.359, M06.361, M06.362, M06.369, M06.371, M06.372, M06.379, M06.38, M06.39, M06.80, M06.811, M06.812, M06.819, M06.821, M06.822, M06.829, M06.831, M06.832, M06.839, M06.841, M06.842, M06.849, M06.851, M06.852, M06.859, M06.861, M06.862, M06.869, M06.871, M06.872, M06.879, M06.88, M06.89, M06.9, M08.00, M08.011, M08.012, M08.019, M08.021, M08.022, M08.029, M08.031, M08.032, M08.039, M08.041, M08.042, M08.049, M08.051, M08.052, M08.059, M08.061, M08.062, M08.069, M08.071, M08.072, M08.079, M08.08, M08.09, M08.1, M08.20, M08.211, M08.212, M08.219, M08.221, M08.222, M08.229, M08.231, M08.232, M08.239, M08.241, M08.242, M08.249, M08.251, M08.252, M08.259, M08.261, M08.262, M08.269, M08.271, M08.272, M08.279, M08.28, M08.29, M08.3, M08.40, M08.411, M08.412, | 208 (10.8) |

**Supplemental Table 1 (continued).** US Dept. of Health and Human Services – 20 Chronic Conditions and associated International Classification of Diseases (ICD) ICD-9 and ICD-10 codes

| **Condition description** | **CCS** | **CMS (ICD-9 Codes)** | **CMS (ICD-10 Codes)** | **Persons with condition at baseline**  **N (%)** |
| --- | --- | --- | --- | --- |
| Arthritis  *(continued)* | --- | --- | M08.419, M08.421, M08.422, M08.429, M08.431, M08.432, M08.439, M08.441, M08.442, M08.449, M08.451, M08.452, M08.459, M08.461, M08.462, M08.469, M08.471, M08.472, M08.479, M08.48, M08.80, M08.811, M08.812, M08.819, M08.821, M08.822, M08.829, M08.831, M08.832, M08.839, M08.841, M08.842, M08.849, M08.851, M08.852, M08.859, M08.861, M08.862, M08.869, M08.871, M08.872, M08.879, M08.88, M08.89, M08.90, M08.911, M08.912, M08.919, M08.921, M08.922, M08.929, M08.931, M08.932, M08.939, M08.941, M08.942, M08.949, M08.951, M08.952, M08.959, M08.961, M08.962, M08.969, M08.971, M08.972, M08.979, M08.98, M08.99, M15.0, M15.1, M15.2, M15.3, M15.4, M15.8, M15.9, M16.0, M16.10, M16.11, M16.12, M16.2, M16.30, M16.31, M16.32, M16.4, M16.50, M16.51, M16.52, M16.6, M16.7, M16.9, M17.0, M17.10, M17.11, M17.12, M17.2, M17.30, M17.31, M17.32,  M17.4, M17.5, M17.9, M18.0, M18.10, M18.11, M18.12, M18.2, M18.30, M18.31, M18.32, M18.4, M18.50, M18.51, M18.52, M18.9, M19.011, M19.012, M19.019, M19.021, M19.022, M19.029, M19.031, M19.032, M19.039, M19.041, M19.042, M19.049, M19.071, M19.072, M19.079, M19.111, M19.112, M19.119, M19.121, M19.122, M19.129, M19.131, M19.132, M19.139, M19.141, M19.142, M19.149, M19.171, M19.172, M19.179, M19.211, M19.212, M19.219, M19.221, M19.222, M19.229, M19.231, M19.232, M19.239, M19.241, M19.242, M19.249, M19.271, M19.272, M19.279, M19.90, M19.91, M19.92, M19.93, M45.0, M45.1, M45.2, M45.3, M45.4, M45.5, M45.6, M45.7, M45.8, M45.9, M47.011, M47.012, M47.013, M47.014, M47.015, M47.016, M47.019, M47.021, M47.022, M47.029, M47.10, M47.11, M47.12, M47.13, M47.20, M47.21, M47.22, M47.23, M47.24, M47.25, M47.26, M47.27, M47.28, M47.811, M47.812, M47.813, M47.814, M47.815, M47.816, M47.817, M47.818, M47.819, M47.891, M47.892, M47.893, M47.894, M47.895, M47.896, M47.897, M47.898, M47.899, M47.9, M48.8X1, M48.8X2, M48.8X3, M48.8X4, M48.8X5, M48.8X6, M48.8X7, M48.8X8, M48.8X9 | 208 (10.8) |
| Asthma | 128 | 493.00, 493.01, 493.02, 493.10, 493.11,  493.12, 493.20, 493.21, 493.22, 493.81,  493.82, 493.90, 493.91, 493.92 | J45.20, J45.21, J45.22, J45.30, J45.31, J45.32, J45.40, J45.41, J45.42, J45.50, J45.51, J45.52, J45.901, J45.902, J45.909, J45.990, J45.991, J45.998 | 5 (0.3) |
| Autism spectrum disorder | ICD-9 codes 299.00,  299.01;  ICD-10 code F84.0 * | Not applicable | Not applicable | 0 (0) |
| Cancer | 11–43 | 140-209, 230-234, 258.02, 258.03, 511.81, 789.51, 795-796, V10.00-V10.91, V71.1 | C00-C26, C30-C34, C37-C41, C43-49, C4A.0-C4A.9, C50-C58, C60-C79, C7A.00-C78.1, C7A.8, C7B.0-C7B.1, C7B.8, C80-C86, C88, C90-C96, D00-D07, D09, E31.22, E31.23, J91.0, R18.0, R85.610-R85.614, R87.610-R87-614, R87.620-R87.624, Z85.01-Z85.12, Z85.2-Z85.9, Z86.00-Z86.01, Z86.03 | 129 (6.7) |
| Chronic kidney disease | 158 | 016.00, 016.01, 016.02, 016.03, 016.04,  016.05, 016.06, 095.4, 189.0, 189.9, 223.0,  236.91, 249.40, 249.41, 250.40, 250.41,  250.42, 250.43, 271.4, 274.10, 283.11,  403.01, 403.11, 403.91, 404.02, 404.03,  404.12, 404.13, 404.92, 404.93, 440.1,  442.1, 572.4, 580.0, 580.4, 580.81, 580.89,  580.9, 581.0, 581.1, 581.2, 581.3, 581.81,  581.89, 581.9, 582.0, 582.1, 582.2, 582.4,  582.81, 582.89, 582.9, 583.0, 583.1, 583.2,  583.4, 583.6, 583.7, 583.81, 583.89, 583.9,  584.5, 584.6, 584.7, 584.8, 584.9, 585.1,  585.2, 585.3, 585.4, 585.5, 585.6, 585.9,  586, 587, 588.0, 588.1, 588.81, 588.89,  588.9, 591, 753.12, 753.13, 753.14, 753.15,  753.16, 753.17, 753.19, 753.20, 753.21,  753.22, 753.23, 753.29, 794.4 | A18.11, A52.75, B52.0, C64.1, C64.2, C64.9, C68.9, D30.00, D30.01, D30.02, D41.00, D41.01, D41.02, D41.10, D41.11, D41.12, D41.20, D41.21, D41.22, D59.3, E08.21, E08.22, E08.29, E08.65, E09.21, E09.22, E09.29, E10.21, E10.22, E10.29, E10.65, E11.21, E11.22, E11.29, E11.65, E13.21, E13.22, E13.29, E74.8, I12.0, I12.9, I13.0, I13.10, I13.11, I13.2, I70.1, I72.2, K76.7, M10.30, M10.311, M10.312, M10.319, M10.321, M10.322, M10.329, M10.331, M10.332, M10.339, M10.341, M10.342, M10.349, M10.351, M10.352, M10.359, M10.361, M10.362, M10.369, M10.371, M10.372, M10.379, M10.38, M10.39, M32.14, M32.15, M35.04, N00.0, N00.1, N00.2, N00.3, N00.4, N00.5, N00.6, N00.7, N00.8, N00.9, N01.0, N01.1, N01.2, N01.3, N01.4,  N01.5, N01.6, N01.7, N01.8, N01.9, N02.0, N02.1, N02.2, N02.3, N02.4, N02.5, N02.6,  N02.7, N02.8, N02.9, N03.0, N03.1, N03.2, N03.3, N03.4, N03.5, N03.6, N03.7, N03.8,  N03.9, N04.0, N04.1, N04.2, N04.3, N04.4, N04.5, N04.6, N04.7, N04.8, N04.9, N05.0,  N05.1, N05.2, N05.3, N05.4, N05.5, N05.6, N05.7, N05.8, N05.9, N06.0, N06.1, N06.2,  N06.3, N06.4, N06.5, N06.6, N06.7, N06.8, N06.9, N07.0, N07.1, N07.2, N07.3, N07.4,  N07.5, N07.6, N07.7, N07.8, N07.9, N08, N13.1, N13.2, N13.30, N13.39, N14.0, N14.1,  N14.2, N14.3, N14.4, N15.0, N15.8, N15.9, N16, N17.0, N17.1, N17.2, N17.8, N17.9,  N18.1, N18.2, N18.3, N18.4, N18.5, N18.6, N18.9, N19, N25.0, N25.1, N25.81, N25.89,  N25.9, N26.1, N26.9, Q61.02, Q61.11, Q61.19, Q61.2, Q61.3, Q61.4, Q61.5, Q61.8, Q62.0, Q62.2, Q62.10, Q62.11, Q62.12, Q62.31, Q62.32, Q62.39, R94.4 | 1 (0.1) |
| Chronic obstructive pulmonary disease | 127 | 490, 491.0, 491.1, 491.20, 491.21, 491.22,  491.8, 491.9, 492.0, 492.8, 494.0, 494.1,  496 | J40, J41.0, J41.1, J41.8, J42, J43.0, J43.1, J43.2, J43.8, J43.9, J44.0, J44.1, J44.9, J47.0, J47.1, J47.9 | 3 (0.2) |
| Dementia (including Alzheimer’s and other senile dementias) | 653 | 331.0, 331.11, 331.19, 331.2, 331.7, 290.0,  290.10, 290.11, 290.12, 290.13, 290.20,  290.21, 290.3, 290.40, 290.41, 290.42,  290.43, 294.0, 294.10, 294.11, 294.8, 797 | F01.50, F01.51, F02.80, F02.81, F03.90, F03.91, F04, G13.8, F05, F06.1, F06.8, G30.0, G30.1, G30.8, G30.9, G31.1, G31.2, G31.01, G31.09, G94, R41.81, R54 | 4 (0.2) |

**Supplemental Table 1 (continued).** US Dept. of Health and Human Services – 20 Chronic Conditions and associated International Classification of Diseases (ICD) ICD-9 and ICD-10 codes

| **Condition description** | **CCS** | **CMS (ICD-9 Codes)** | **CMS (ICD-10 Codes)** | **Persons with condition at baseline**  **N (%)** |
| --- | --- | --- | --- | --- |
| Depression | 657 | 296.20, 296.21, 296.22, 296.23, 296.24,  296.25, 296.26, 296.30, 296.31, 296.32,  296.33, 296.34, 296.35, 296.36, 296.51,  296.52, 296.53, 296.54, 296.55, 296.56,  296.60, 296.61, 296.62, 296.63, 296.64,  296.65, 296.66, 296.89, 298.0, 300.4, 309.1,  311 | F31.30, F31.31, F31.32, F31.4, F31.5, F31.60, F31.61, F31.62, F31.63, F31.64, F31.75, F31.76, F31.77, F31.78, F31.81, F32.0, F32.1, F32.2, F32.3, F32.4, F32.5, F32.9, F33.0, F33.1, F33.2, F33.3, F33.40, F33.41, F33.42, F33.8, F33.9, F34.1, F43.21, F43.23 | 5 (0.3) |
| Diabetes | 49, 50 | 249.00, 249.01, 249.10, 249.11, 249.20,  249.21, 249.30, 249.31, 249.40, 249.41,  249.50, 249.51, 249.60, 249.61, 249.70,  249.71, 249.80, 249.81, 249.90, 249.91,  250.00, 250.01, 250.02, 250.03, 250.10,  250.11, 250.12, 250.13, 250.20, 250.21,  250.22, 250.23, 250.30, 250.31, 250.32,  250.33, 250.40, 250.41, 250.42, 250.43,  250.50, 250.51, 250.52, 250.53, 250.60,  250.61, 250.62, 250.63, 250.70, 250.71,  250.72, 250.73, 250.80, 250.81, 250.82,  250.83, 250.90, 250.91, 250.92, 250.93,  357.2, 362.01, 362.02, 362.03, 362.04,  362.05, 362.06, 366.41 | E08.00, E08.01, E08.10, E08.11, E08.21, E08.22, E08.29, E08.311, E08.319, E08.321, E08.3211, E08.3212, E08.3213, E08.3219, E08.329, E08.3291, E08.3292, E08.3293, E08.3299, E08.331, E08.3311, E08.3312, E08.3313, E08.3319, E08.339, E08.3391, E08.3392, E08.3393, E08.3399, E08.341, E08.3411, E08.3412, E08.3413, E08.3419, E08.349, E08.3491, E08.3492, E08.3493, E08.3499, E08.351, E08.3511, E08.3512, E08.3513, E08.3519, E08.3521, E08.3522, E08.3523, E08.3529, E08.3531, E08.3532, E08.3533, E08.3539, E08.3541, E08.3542, E08.3543, E08.3549, E08.3551, E08.3552, E08.3553, E08.3559, E08.359, E08.3591, E08.3592, E08.3593, E08.3599, E08.36, E08.37X1, E08.37X2, E08.37X3, E08.37X9, E08.39, E08.40, E08.41, E08.42, E08.43, E08.44, E08.49, E08.51, E08.52, E08.59, E08.610, E08.618, E08.620, E08.621, E08.622, E08.628, E08.630, E08.638, E08.641, E08.649, E08.65, E08.69, E08.8, E08.9, E09.00, E09.01, E09.10, E09.11, E09.21, E09.22, E09.29, E09.311, E09.319, E09.321, E09.3211, E09.3212, E09.3213, E09.3219, E09.329, E09.3291, E09.3292, E09.3293, E09.3299, E09.331, E09.3311, E09.3312, E09.3313, E09.3319, E09.339, E09.3391, E09.3392, E09.3393, E09.3399, E09.341, E09.3411, E09.3412, E09.3413, E09.3419, E09.349, E09.3491, E09.3492, E09.3493, E09.3499, E09.351, E09.3511, E09.3512, E09.3513, E09.3519, E09.3521, E09.3522, E09.3523, E09.3529, E09.3531, E09.3532, E09.3533, E09.3539, E09.3541, E09.3542, E09.3543, E09.3549, E09.3551, E09.3552, E09.3553, E09.3559, E09.359, E09.3591, E09.3592, E09.3593, E09.3599, E09.36, E09.37X1, E09.37X2, E09.37X3, E09.37X9, E09.39, E09.40, E09.41, E09.42, E09.43, E09.44, E09.49, E09.51, E09.52, E09.59, E09.610, E09.618, E09.620, E09.621, E09.622, E09.628, E09.630, E09.638, E09.641, E09.649, E09.65, E09.69, E09.8, E09.9, E10.10, E10.11, E10.21, E10.22, E10.29, E10.311, E10.319, E10.321, E10.3211, E10.3212, E10.3213, E10.3219, E10.329, E10.3291, E10.3292, E10.3293, E10.3299, E10.331, E10.3311, E10.3312, E10.3313, E10.3319, E10.339, E10.3391, E10.3392, E10.3393, E10.3399, E10.341, E10.3411, E10.3412, E10.3413, E10.3419, E10.349, E10.3491, E10.3492, E10.3493, E10.3499, E10.351, E10.3511, E10.3512, E10.3513, E10.3519, E10.359, E10.36, E10.37X1, E10.37X2, E10.37X3, E10.37X9, E10.39, E10.40, E10.41, E10.42, E10.43, E10.44, E10.49, E10.51, E10.52, E10.59, E10.610, E10.618, E10.620, E10.621, E10.622, E10.628, E10.630, E10.638, E10.641, E10.649, E10.65, E10.69, E10.8, E10.9, E11.00, E11.01, E11.10, E11.11, E11.21, E11.22, E11.29, E11.311, E11.319, E11.321, E11.3211, E11.3212, E11.3213, E11.3219, E11.329, E11.3291, E11.3292, E11.3293, E11.3299, E11.331, E11.3311, E11.3312, E11.3313, E11.3319, E11.339, E11.3391, E11.3392, E11.3393, E11.3399, E11.341, E11.3411, E11.3412, E11.3413, E11.3419, E11.349, E11.3491, E11.3492, E11.3493, E11.3499, E11.351, E11.3511, E11.3512, E11.3513, E11.3519, E11.3521, E11.3522, E11.3523, E11.3529, E11.3531, E11.3532, E11.3533, E11.3539, E11.3541, E11.3542, E11.3543, E11.3549, E11.3551, E11.3552, E11.3553, E11.3559, E11.359, E11.3591, E11.3592, E11.3593, E11.3599, E11.36, E11.37X1, E11.37X2, E11.37X3, E11.37X9, E11.39, E11.40, E11.41, E11.42, E11.43, E11.44, E11.49, E11.51, E11.52, E11.59, E11.610, E11.618, E11.620, E11.621, E11.622, E11.628, E11.630, E11.638, E11.641, E11.649, E11.65, E11.69, E11.8, E11.9, E13.00, E13.01, E13.10, E13.11, E13.21, E13.22, E13.29, E13.311, E13.319, E13.321, E13.3211, E13.3212, E13.3213, E13.3219, E13.329, E13.3291, E13.3292, E13.3293, E13.3299, E13.331, E13.3311, E13.3312, E13.3313, E13.3319, E13.339, E13.3391, E13.3392, E13.3393, E13.3399, E13.341, E13.3411, E13.3412, E13.3413, E13.3419, E13.349, E13.3491, E13.3492, E13.3493, E13.3499, E13.351, E13.3511, E13.3512, E13.3513, E13.3519, E13.3521, E13.3522, E13.3523, E13.3529, E13.3531, E13.3532, E13.3533, E13.3539, E13.3541, E13.3542, E13.3543, E13.3549, E13.3551, E13.3552, E13.3553, E13.3559, E13.359, E13.36, E13.39, E13.40, E13.41, E13.42, E13.43, E13.44, E13.49, E13.51, E13.52, E13.59, E13.610, E13.618, E13.620, E13.621, E13.622, E13.628, E13.630, E13.638, E13.641, E13.649, E13.65, E13.69, E13.8, E13.9 | 23 (1.2) |
| Hepatitis | 6 | Not applicable | Not applicable | 1 (0.1) |
| Human immunodeficiency virus (HIV) | 5 | Not applicable | Not applicable | 0 (0) |
| Osteoporosis | 206 | 733.00, 733.01, 733.02, 733.03, 733.09 | M81.0, M81.6, M81.8 | 41 (2.1) |
| Schizophrenia | 659 | Not applicable | Not applicable | 0 (0) |
| Substance abuse disorders (drug and alcohol) | 660, 661 | Not applicable | Not applicable | 0 (0) |

- Autism spectrum disorder values listed in the CCS column are ICD-9 and ICD-10 codes, not CCS chapter numbers.

# Notes:

- 1. The 20 chronic conditions are defined by the U.S. Department of Health and Human Services as detailed elsewhere [(http://www.cdc.gov/pcd/issues/2013/12_0239.htm](http://www.cdc.gov/pcd/issues/2013/12_0239.htm)).
  2. Each CCS category includes a list of ICD-9 and ICD-10 codes as detailed elsewhere [(https://www.ccwdata.org/web/guest/condition-categories](https://www.ccwdata.org/web/guest/condition-categories)). Some of the CCS lists of codes are extensive and not reproduced here (e.g., for cancers).

**Supplemental Table 2.** Senescence-associated protein names and aliases

| **Biomarker** | **Protein full name** | **Alias** |
| --- | --- | --- |
| Activin A | Activin A | INHBA |
| ADAMTS13 | A disintegrin and metalloproteinase with thrombospondin motifs 13 | VWFCP |
| Eotaxin | Eotaxin | CCL11 |
| Fas | Tumor necrosis factor receptor superfamily member 6 | APT1, TNFRSF6 |
| GDF15 | Growth/differentiation factor 15 | MIC1, NAG1, NRG1 |
| Gro-alpha | Chemokine growth-regulated protein alpha | CXCL1 |
| ICAM1 | Intercellular adhesion molecule 1 | CD54 |
| IL7 | Interleukin 7 |  |
| MCP1 | Monocyte chemotactic protein 1 | CCL2 |
| MDC | Macrophage-derived chemokine | CCL22, SCYA22 |
| MMP1 | Matrix metalloproteinase 1 | Interstitial collagenase |
| MMP2 | Matrix metalloproteinase 2 | CLG4A |
| MMP7 | Matrix metalloproteinase 7 | Matrilysin |
| MMP9 | Matrix metalloproteinase 9 | CLG4B |
| MPO | Myeloperoxidase |  |
| OPN | Osteopontin | SPP1 |
| PAI1 | Plasminogen activator inhibitor 1 | SERPINE1, PLANH1 |
| PARC | Pulmonary and activation-regulated chemokine | CCL18 |
| PDGF-AA | Platelet-derived growth factor alpha polypeptide a | PDGF-1 |
| PDGF-AB | Platelet-derived growth factor alpha polypeptide b | PDGF-2 |
| RAGE | Advanced glycosylation end product-specific receptor | AGER |
| RANTES | Regulated on Activation, Normal T Cell Expressed and Secreted | CCL5, SCYA5 |
| SOST | Sclerostin | DAND6 |
| TNFα | Tumor necrosis factor alpha | TNFSF2 |
| TNFR1 | Tumor necrosis factor receptor 1 | TNFRSF1A, CD120a |
| TNFR2 | Tumor necrosis factor receptor 2 | TNFRSF1B |
| uPAR | Urokinase-type plasminogen activator receptor | PLAUR |
| VEGFA | Vascular endothelial growth factor A | VPF |

**Supplemental Table 3.**  Spearman correlation coefficients between standardized senescent biomarker concentrations and participant chronological age.

| **Biomarker** | ***r*** | **P** |
| --- | --- | --- |
| Activin A | 0.30 | <0.001 |
| ADAMTS13 | -0.07 | 0.001 |
| Eotaxin | 0.02 | 0.33 |
| Fas | 0.19 | <0.001 |
| GDF15 | 0.39 | <0.001 |
| Gro-alpha | 0.09 | <0.001 |
| ICAM1 | 0.09 | <0.001 |
| IL7 | -0.07 | 0.004 |
| MCP1 | 0.06 | 0.007 |
| MDC | 0.08 | <0.001 |
| MMP1 | 0.04 | 0.10 |
| MMP2 | 0.20 | <0.001 |
| MMP7 | 0.16 | <0.001 |
| MMP9 | 0.01 | 0.53 |
| MPO | 0.09 | <0.001 |
| OPN | 0.13 | <0.001 |
| PAI1 | -0.09 | <0.001 |
| PARC | 0.16 | <0.001 |
| PDGF-AA | -0.04 | 0.07 |
| PDGF-AB | -0.07 | 0.003 |
| RAGE | 0.09 | <0.001 |
| RANTES | -0.09 | <0.001 |
| SOST | -0.01 | 0.60 |
| TNFα | 0.13 | <0.001 |
| TNFR1 | 0.26 | <0.001 |
| TNFR2 | 0.23 | <0.001 |
| uPAR | 0.15 | <0.001 |
| VEGFA | 0.09 | <0.001 |

**Supplemental Table 4.** Plasma concentrations of senescence biomarkers in women and men.

| **Biomarker** | **Men**  Median (IQR) | **Women**  Median (IQR) | **P** |
| --- | --- | --- | --- |
| Activin A | 276.3 (226.5, 334.6) | 261.5 (210.5, 322.0) | 0.002 |
| ADAMTS13 | 910266.9 (663048.0, 1167644.9) | 891746.2 (658477.5, 1131509.2) | 0.33 |
| Eotaxin | 119.6 (95.5, 161.2) | 118.6 (90.6, 161.6) | 0.30 |
| Fas | 6388.0 (5306.5, 7602.7) | 6051.1 (5093.8, 7159.0) | <0.001 |
| GDF15 | 851.8 (634.7, 1211.5) | 744.8 (545.7, 998.1) | <0.001 |
| Gro-alpha | 101.1 (83.7, 128.0) | 101.1 (79.6, 125.7) | 0.61 |
| ICAM1 | 306140.3 (232818.1, 403032.4) | 281291.3 (213573.5, 358535.6) | <0.001 |
| IL7 | 1.8 (1.0, 2.9) | 1.7 (1.0, 2.8) | 0.85 |
| MCP1 | 145.3 (116.0, 180.6) | 143.2 (116.1, 183.2) | 0.62 |
| MDC | 297.7 (219.9, 394.9) | 350.8 (261.7, 460.4) | <0.001 |
| MMP1 | 754.2 (473.1, 1196.3) | 779.4 (501.1, 1319.4) | 0.08 |
| MMP2 | 370412.7 (321470.9, 426927.5) | 372346.2 (316848.0, 427016.3) | 0.45 |
| MMP7 | 2284.9 (1558.3, 3571.5) | 2359.9 (1594.4, 3501.7) | 0.15 |
| MMP9 | 60989.4 (42316.4, 90127.4) | 50366.1 (36634.5, 72027.9) | <0.001 |
| MPO | 23016.1 (18601.6, 29808.9) | 24098.2 (19193.3, 30484.7) | 0.15 |
| OPN | 34117.0 (24715.2, 45275.2) | 33654.9 (24534.0, 44450.1) | 0.18 |
| PAI1 | 21998.6 (14447.2, 32606.5) | 22790.9 (15046.2, 34358.5) | 0.12 |
| PARC | 49818.1 (36587.0, 71304.4) | 46275.0 (34880.4, 64546.4) | 0.04 |
| PDGF-AA | 1571.5 (920.9, 2477.8) | 1658.4 (993.5, 2613.4) | 0.13 |
| PDGF-AB | 567.5 (319.3, 980.7) | 583.7 (324.0, 971.5) | 0.99 |
| RAGE | 1625.9 (1257.8, 2125.2) | 1752.5 (1316.2, 2262.6) | <0.001 |
| RANTES | 21967.4 (11759.8, 35051.4) | 24329.6 (13679.5, 39015.4) | 0.002 |
| SOST | 627.2 (468.6, 831.8) | 518.0 (379.4, 663.7) | <0.001 |
| TNFα | 3.6 (2.7, 4.6) | 3.3 (2.4, 4.5) | 0.046 |
| TNFR1 | 1106.0 (903.6, 1345.4) | 1001.4 (839.5, 1275.2) | <0.001 |
| TNFR2 | 2939.7 (2265.8, 3942.4) | 2682.9 (2076.9, 3556.0) | 0.007 |
| uPAR | 433.1 (318.6, 554.8) | 472.3 (351.9, 628.5) | <0.001 |
| VEGFA | 51.4 (35.8, 73.0) | 50.3 (34.5, 72.3) | 0.52 |

Abbreviations: IQR, interquartile range.

**Supplemental Table 5**: Hazard ratios (HR) for death for variables selected using LASSO regression

| **Characteristic** | **Potential predictors include age, sex, race, and number of prior conditions** | **Potential predictors include all biomarkers*** | **Potential predictors include all biomarkers* with age, sex, race, and number of prior conditions forced in** |
| --- | --- | --- | --- |
|  | **HR** | **HR** | **HR** |
| Age (10-year increase) | 2.69 | - | 1.76 |
| Sex (women vs. men) | 0.55 | - | 0.61 |
| Race (white vs. non-white) | 1.24 | - | 1.16 |
| 1 vs 0 chronic conditions | 0.49 | - | 0.56 |
| GDF15 | - | 2.03 | 1.79 |
| RAGE | - | 0.77 | 0.81 |
| VEGFA | - | 1.19 | 1.21 |
| PARC | - | 1.17 | 1.14 |
| MMP2 | - | 1.18 | 1.13 |
| PAI1 | - | 0.86 | 0.92 |
| TNFR1 | - | 1.16 | 1.09 |
| Fas | - | 0.91 | 0.91 |
| MMP9 | - | 0.97 | 0.92 |
| MMP7 | - | 1.06 | 1.05 |
| OPN | - | 1.07 | 1.03 |
| ICAM1 | - | 0.98 | 0.97 |
| RANTES | - | - | 0.96 |
| TNFα | - | - | 1.03 |
| MCP1 | - | 0.99 | - |
| MDC | - | 0.99 | - |
| C-statistic (95% CI) |  |  |  |
| Training dataset | 0.70 (0.67, 0.74) | 0.76 (0.73, 0.80) | 0.79 (0.76, 0.82)^**^ |
| Testing dataset | 0.68 (0.60, 0.75) | 0.70 (0.63, 0.77) | 0.72 (0.66, 0.79)^+^ |

*SASP proteins are log transformed and standardized to mean=0, standard deviation=1.

^**^P value<0.001 for the difference in c-statistics between the model including age, sex, race, and number of prior conditions and the final full model (training dataset)

^+^P value=0.13 for the difference in c-statistics between the model including age, sex, race, and number of prior conditions and the final full model (test dataset)

**Supplemental Table 6**: Hazard ratios (HR) for the associations between baseline characteristics, biomarkers, and death using both LASSO regression and Cox proportional hazards modelling.

|  | **LASSO** | **COX** | **Model with only GDF15** | **Model with GDF15 and RAGE** |
| --- | --- | --- | --- | --- |
| **Characteristic** | **HR** | **HR (95% CI)** | **HR (95% CI)** | **HR (95% CI)** |
| Age (10-year increase) | 1.76 | 1.68 (1.37, 2.07) | 1.83 (1.49, 2.24) | 1.87 (1.52, 2.29) |
| Sex (women vs. men) | 0.61 | 0.64 (0.50, 0.82) | 0.60 (0.47, 0.76) | 0.63 (0.50, 0.80) |
| Race (white vs. non-white) | 1.16 | 1.76 (0.78, 4.02) | 1.23 (0.58, 2.62) | 1.29 (0.61, 2.74) |
| 1 vs 0 chronic conditions | 0.56 | 0.56 (0.42, 0.73) | 0.54 (0.41, 0.71) | 0.56 (0.42, 0.73) |
| GDF15 | 1.79 | 1.63 (1.41, 1.89) | 1.80 (1.61, 2.01) | 1.86 (1.66, 2.08) |
| RAGE | 0.81 | 0.71 (0.61, 0.82) | - | 0.81 (0.72, 0.91) |
| VEGFA | 1.21 | 1.33 (1.16, 1.52) | - | - |
| PARC | 1.14 | 1.27 (1.12, 1.44) | - | - |
| MMP2 | 1.13 | 1.20 (1.06, 1.36) | - | - |
| PAI1 | 0.92 | 0.84 (0.69, 1.01) | - | - |
| TNFR1 | 1.09 | 1.16 (0.94, 1.42) | - | - |
| Fas | 0.91 | 0.89 (0.75, 1.07) | - | - |
| MMP9 | 0.92 | 0.92 (0.81, 1.05) | - | - |
| MMP7 | 1.05 | 1.04 (0.90, 1.21) | - | - |
| OPN | 1.03 | 1.08 (0.94, 1.24) | - | - |
| ICAM1 | 0.97 | 0.95 (0.85, 1.07) | - | - |
| RANTES | 0.96 | 0.92 (0.77, 1.09) | - | - |
| TNFα | 1.03 | 1.04 (0.91, 1.20) | - | - |
| C-statistic (95% CI) | 0.79 (0.73, 0.80) | 0.76 (0.73, 0.80) | 0.74 (0.70, 0.78) | 0.74 (0.69, 0.79) |

**Supplemental Table 7**. Assay performance characteristics, including measurement range, sensitivity, and the coefficient of variation (CV) for biomarkers of cellular senescence.

| **Biomarker** | **Range (pg/ml)*** | **Sensitivity (pg/ml)** | **CV (%)** |
| --- | --- | --- | --- |
| Activin A | 15.6-1000 | 7.85 | 5.95 |
| ADAMTS13 | 16000-3900000 | 1270 | 20.21 |
| Eotaxin | 32.3-23580 | 1.81 | 10.46 |
| Fas | 144-35000 | 3.2 | 10.81 |
| GDF15 | 18.5-4500 | 1.2 | 16.61 |
| Gro-alpha | 49.8-12000 | 5.3 | 17.06 |
| ICAM1 | 7000-1700000 | 87.9 | 20.21 |
| IL7 | 0.9-3620 | 0.140 | 12.30 |
| MCP1 | 4.2-3070 | 0.6 | 5.19 |
| MDC | 53.5-13000 | 8.5 | 11.03 |
| MMP1 | 49.4-12000 | 2.7 | 28.12 |
| MMP2 | 276-67000^*^ | 108 | 13.69 |
| MMP7 | 226-55000 | 23.2 | 25.92 |
| MMP9 | 123-30000^*^ | 13.6 | 6.86 |
| MPO | 123-30000^*^ | 26.2 | 10.42 |
| OPN | 1440-350000 | 413 | 14.75 |
| UPAR | 165-40000 | 43.2 | 20.19 |
| PAI1 | 18.1-4400^*^ | 0.7 | 8.05 |
| PARC | 18.5-4500^*^ | 0.3 | 9.08 |
| PDGF-AA | 6.6-4800 | 0.86 | 14.63 |
| PDGF-AB | 6.9-5000 | 0.23 | 8.02 |
| RAGE | 123-30000 | 7.2 | 10.04 |
| RANTES | 20.6-5000^*^ | 1.8 | 25.95 |
| SOST | 10.3-2500 | 7 | 26.57 |
| TNFα | .757-3100 | 0.54 | 12.17 |
| TNFR1 | 49.4-12000 | 41 | 12.36 |
| TNFR2 | 11.1-2700 | 0.5 | 41.96 |
| VEGFA | 7-4280 | 1.17 | 22.34 |

^*^ Denotes biomarkers analyzed in plasma samples at 1:100 dilution. All other biomarkers analyzed in plasma samples at 1:2 dilution.

**Supplemental Table 8.**  Spearman correlation coefficients between standardized senescent biomarker concentrations.

|  | **ADAMTS13** | **Eotaxin** | **Fas** | **GDF 15** | **Gro-alpha** | **ICAM1** | **IL7** | **MCP1** | **MDC** | **MMP1** | **MMP2** | **MMP7** | **MMP9** | **MPO** | **OPN** |
| --- | --- | --- | --- | --- | --- | --- | --- | --- | --- | --- | --- | --- | --- | --- | --- |
| **Activin A** | -0.05^*^ | 0.07^*^ | 0.36^*^ | 0.39^*^ | 0.15^*^ | 0.18^*^ | 0.04 | 0.20^*^ | 0.16^*^ | 0.09^*^ | 0.36^*^ | 0.19^*^ | 0.06^*^ | 0.21^*^ | 0.14^*^ |
| **ADAMTS13** |  | 0.01 | 0.03 | -0.06^*^ | -0.01 | 0.04 | 0.05^*^ | 0.09^*^ | 0.06^*^ | 0.04 | -0.01 | 0.30^*^ | -0.003 | -0.02 | 0.15^*^ |
| **Eotaxin** |  |  | 0.09^*^ | 0.08^*^ | 0.19^*^ | 0.07^*^ | 0.27^*^ | 0.41^*^ | 0.12^*^ | 0.41^*^ | 0.06^*^ | 0.04 | 0.25^*^ | 0.10^*^ | 0.02 |
| **Fas** |  |  |  | 0.38^*^ | 0.21^*^ | 0.27^*^ | -0.01 | 0.16^*^ | 0.16^*^ | 0.06^*^ | 0.24^*^ | 0.17* | 0.03 | 0.12^*^ | 0.19^*^ |
| **GDF15** |  |  |  |  | 0.27^*^ | 0.25^*^ | -0.01 | 0.16^*^ | 0.19^*^ | 0.13^*^ | 0.19^*^ | 0.22^*^ | 0.05^*^ | 0.14^*^ | 0.18^*^ |
| **Gro-alpha** |  |  |  |  |  | 0.43^*^ | 0.33^*^ | 0.09^*^ | 0.21^*^ | 0.38^*^ | 0.11^*^ | -0.02 | 0.06^*^ | 0.11* | 0.05^*^ |
| **ICAM1** |  |  |  |  |  |  | -0.03 | 0.07^*^ | 0.16^*^ | 0.13^*^ | 0.10^*^ | 0.06^*^ | 0.07^*^ | 0.16^*^ | 0.09^*^ |
| **IL7** |  |  |  |  |  |  |  | 0.09^*^ | 0.13^*^ | 0.42* | -0.02 | 0.01 | 0.07^*^ | 0.11^*^ | -0.04 |
| **MCP1** |  |  |  |  |  |  |  |  | 0.14^*^ | 0.24^*^ | 0.08^*^ | 0.21^*^ | 0.14^*^ | 0.11^*^ | 0.11^*^ |
| **MDC** |  |  |  |  |  |  |  |  |  | 0.15^*^ | 0.07^*^ | 0.14^*^ | 0.02 | 0.16^*^ | 0.10^*^ |
| **MMP1** |  |  |  |  |  |  |  |  |  |  | -0.01 | 0.10^*^ | 0.17^*^ | 0.19^*^ | 0.03 |
| **MMP2** |  |  |  |  |  |  |  |  |  |  |  | 0.02 | 0.01 | 0.14^*^ | 0.15^*^ |
| **MMP7** |  |  |  |  |  |  |  |  |  |  |  |  | -0.04 | 0.10^*^ | 0.23^*^ |
| **MMP9** |  |  |  |  |  |  |  |  |  |  |  |  |  | 0.38^*^ | -0.05^*^ |
| **MPO** |  |  |  |  |  |  |  |  |  |  |  |  |  |  | 0.10^*^ |

^*^P<0.05

**Supplemental Table 8 (continued).**  Spearman correlation coefficients between standardized senescent biomarker concentrations.

|  | **PAI1** | **PARC** | **PDGF-AA** | **PDGF-AB** | **RAGE** | **RANTES** | **SOST** | **TNFα** | **TNFR1** | **TNFR2** | **uPAR** | **VEGFA** |
| --- | --- | --- | --- | --- | --- | --- | --- | --- | --- | --- | --- | --- |
| **Activin A** | 0.15^*^ | 0.25^*^ | 0.02 | 0.01 | 0.22^*^ | 0.004 | 0.10^*^ | 0.24^*^ | 0.44^*^ | 0.36^*^ | 0.22^*^ | 0.19^*^ |
| **ADAMTS13** | -0.01 | -0.01 | 0.02 | -0.01 | 0.02 | 0.01 | 0.18^*^ | 0.06^*^ | 0.02 | 0.02 | 0.02 | 0.08^*^ |
| **Eotaxin** | 0.46^*^ | 0.06^*^ | 0.58^*^ | 0.52^*^ | 0.04 | 0.36^*^ | 0.51^*^ | 0.10^*^ | 0.09^*^ | 0.03 | 0.14^*^ | 0.48^*^ |
| **Fas** | 0.05^*^ | 0.19^*^ | -0.05^*^ | -0.05^*^ | 0.45^*^ | -0.06^*^ | 0.10^*^ | 0.27^*^ | 0.68^*^ | 0.46^*^ | 0.33^*^ | 0.23^*^ |
| **GDF15** | 0.02 | 0.27^*^ | -0.05^*^ | -0.02 | 0.16^*^ | -0.02 | 0.06^*^ | 0.29^*^ | 0.54^*^ | 0.47^*^ | 0.37^*^ | 0.16^*^ |
| **Gro-alpha** | 0.29^*^ | 0.12^*^ | 0.27^*^ | 0.32^*^ | 0.08^*^ | 0.35^*^ | -0.01 | 0.11^*^ | 0.25^*^ | 0.17^*^ | 0.33^*^ | 0.20^*^ |
| **ICAM1** | 0.05^*^ | 0.15^*^ | -0.02 | 0.001 | 0.12^*^ | -0.03 | 0.06^*^ | 0.21^*^ | 0.30^*^ | 0.27^*^ | 0.35^*^ | 0.12^*^ |
| **IL7** | 0.57^*^ | 0.07^*^ | 0.67^*^ | 0.68^*^ | -0.02 | 0.76^*^ | -0.01 | 0.09^*^ | -0.005 | -0.02 | 0.11^*^ | 0.31^*^ |
| **MCP1** | 0.29^*^ | 0.19^*^ | 0.28^*^ | 0.21^*^ | 0.07^*^ | 0.04 | 0.06^*^ | 0.25^*^ | 0.23^*^ | 0.16^*^ | 0.13^*^ | 0.38^*^ |
| **MDC** | 0.17^*^ | 0.16^*^ | 0.15^*^ | 0.14^*^ | 0.15^*^ | 0.16^*^ | 0.04 | 0.22^*^ | 0.19^*^ | 0.24* | 0.23^*^ | 0.17^*^ |
| **MMP1** | 0.49^*^ | 0.16^*^ | 0.56^*^ | 0.52^*^ | 0.03 | 0.47^*^ | -0.02 | 0.14^*^ | 0.21^*^ | 0.13^*^ | 0.19^*^ | 0.47^*^ |
| **MMP2** | -0.06^*^ | 0.09* | -0.06^*^ | -0.05^*^ | 0.27^*^ | 0.001 | 0.06^*^ | 0.04 | 0.20^*^ | 0.17^*^ | 0.20^*^ | <0.001 |
| **MMP7** | 0.05^*^ | 0.17^*^ | 0.02 | -0.05^*^ | 0.14^*^ | -0.002 | 0.19^*^ | 0.19^*^ | 0.30^*^ | 0.26^*^ | 0.07^*^ | 0.23^*^ |
| **MMP9** | 0.25^*^ | 0.18^*^ | 0.26^*^ | 0.25^*^ | -0.09^*^ | 0.09^*^ | 0.12^*^ | 0.08^*^ | 0.10^*^ | 0.06^*^ | 0.07^*^ | 0.22^*^ |
| **MPO** | 0.22^*^ | 0.33^*^ | 0.15^*^ | 0.10^*^ | 0.01 | 0.08^*^ | 0.04 | 0.26^*^ | 0.29^*^ | 0.27^*^ | 0.18^*^ | 0.27^*^ |
| **OPN** | -0.08^*^ | 0.12^*^ | -0.06^*^ | -0.08^*^ | 0.15^*^ | -0.10^*^ | 0.07^*^ | 0.18^*^ | 0.30^*^ | 0.30^*^ | 0.22^*^ | 0.10^*^ |
| **PAI1** |  | 0.19^*^ | 0.74^*^ | 0.74^*^ | -0.06^*^ | 0.66^*^ | <0.001 | 0.10^*^ | 0.08^*^ | 0.03 | 0.12^*^ | 0.50^*^ |
| **PARC** |  |  | 0.07^*^ | 0.05^*^ | -0.002 | 0.08^*^ | 0.01 | 0.27^*^ | 0.37^*^ | 0.34^*^ | 0.16^*^ | 0.24^*^ |
| **PDGF-AA** |  |  |  | 0.87^*^ | -0.03 | 0.73^*^ | 0.004 | 0.01 | -0.02 | -0.04 | 0.08^*^ | 0.53^*^ |
| **PDGF-AB** |  |  |  |  | -0.01 | 0.77^*^ | 0.004 | -0.01 | -0.05^*^ | -0.04 | 0.11 | 0.43^*^ |
| **RAGE** |  |  |  |  |  | -0.01 | 0.08^*^ | 0.08^*^ | 0.36^*^ | 0.26^*^ | 0.16^*^ | 0.09^*^ |
| **RANTES** |  |  |  |  |  |  | -0.02 | -0.06^*^ | -0.05^*^ | -0.05^*^ | 0.09^*^ | 0.33^*^ |
| **SOST** |  |  |  |  |  |  |  | 0.05^*^ | 0.08^*^ | 0.05^*^ | 0.05^*^ | 0.04 |
| **TNFα** |  |  |  |  |  |  |  |  | 0.44^*^ | 0.43^*^ | 0.25^*^ | 0.28^*^ |
| **TNFR1** |  |  |  |  |  |  |  |  |  | 0.71^*^ | 0.38^*^ | 0.41^*^ |
| **TNFR2** |  |  |  |  |  |  |  |  |  |  | 0.36^*^ | 0.26^*^ |
| **uPAR** |  |  |  |  |  |  |  |  |  |  |  | 0.13^*^ |

^*^P<0.05
